# Impact of payments for environmental services and protected areas on local livelihoods and forest conservation in northern Cambodia

**DOI:** 10.1111/cobi.12423

**Published:** 2014-12-09

**Authors:** Tom Clements, E J Milner-Gulland

**Affiliations:** * Wildlife Conservation Society2300 Southern Boulevard, Bronx, NY, 10460, U.S.A.; † Department of Zoology, University of CambridgeDowning Street, Cambridge, CB2 3EJ, United Kingdom; ‡ Department of Life Sciences and Centre for Environmental Policy, Imperial College LondonSilwood Park Campus, Buckhurst Road, Ascot, SL5 7PY, United Kingdom

**Keywords:** biodiversity conservation, effectiveness, impact evaluation, poverty, well-being, bienestar, conservación de la biodiversidad, efectividad, evaluación de impacto, pobreza

## Abstract

The potential impacts of payments for environmental services (PES) and protected areas (PAs) on environmental outcomes and local livelihoods in developing countries are contentious and have been widely debated. The available evidence is sparse, with few rigorous evaluations of the environmental and social impacts of PAs and particularly of PES. We measured the impacts on forests and human well-being of three different PES programs instituted within two PAs in northern Cambodia, using a panel of intervention villages and matched controls. Both PES and PAs delivered additional environmental outcomes relative to the counterfactual: reducing deforestation rates significantly relative to controls. PAs increased security of access to land and forest resources for local households, benefiting forest resource users but restricting households’ ability to expand and diversify their agriculture. The impacts of PES on household well-being were related to the magnitude of the payments provided. The two higher paying market-linked PES programs had significant positive impacts, whereas a lower paying program that targeted biodiversity protection had no detectable effect on livelihoods, despite its positive environmental outcomes. Households that signed up for the higher paying PES programs, however, typically needed more capital assets; hence, they were less poor and more food secure than other villagers. Therefore, whereas the impacts of PAs on household well-being were limited overall and varied between livelihood strategies, the PES programs had significant positive impacts on livelihoods for those that could afford to participate. Our results are consistent with theories that PES, when designed appropriately, can be a powerful new tool for delivering conservation goals whilst benefiting local people.

El Impacto de los Pagos por Servicios Ambientales y Áreas Protegidas sobre la Subsistencia Local y la Conservación del Bosque en el Norte de Camboya

**Resumen:**

Los impactos potenciales de los pagos por servicios ambientales (PSA) y áreas protegidas (APs) sobre los resultados ambientales y las subsistencias locales en los países en desarrollo son polémicos y se han debatido ampliamente. La evidencia disponible es escasa; ha habido pocas evaluaciones rigurosas de los impactos ambientales y sociales de las APs y particularmente los PSA. Medimos el impacto sobre los bosques y el bienestar humano en tres diferentes programas de PSA que se llevan a cabo dentro de dos APs en el norte de Camboya usando un panel de aldeas de intervención y controles emparejados. Tanto los PSA como las APs brindaron resultados ambientales adicionales en relación a los contrafácticos, esto quiere decir que redujeron las tasas de deforestación significativamente en relación a los controles. Las áreas protegidas incrementaron el acceso seguro a los recursos del suelo y el bosque para las viviendas locales, beneficiando a los usuarios de los recursos del bosque pero restringiendo la habilidad de las viviendas para expandirse y diversificar su agricultura. Los impactos de los pagos por servicios ambientales sobre el bienestar de las viviendas estuvieron relacionados con la magnitud de los pagos proporcionados. Los dos programas de PSA de mayor paga y con conexión al mercado tuvieron impactos positivos significativos, mientras que un programa de menor paga con el objetivo de proteger a la biodiversidad no tuvo un efecto detectable sobre las viviendas, a pesar de sus resultados ambientales positivos. Las viviendas que se inscribieron a los programas de PSA con mayor paga, sin embargo, necesitaban típicamente más bienes capitales, por lo que eran menos pobres y tenían mayor seguridad alimentaria que otros aldeanos. Por esto, mientras los impactos de las APs sobre el bienestar de las viviendas fueron limitados en general y variaron dependiendo de las estrategias de subsistencia, los programas de PSA tuvieron impactos positivos significativos sobre las viviendas para aquellos que podían costear participar. Nuestros resultados son congruentes con las teorías de que los PSA, cuando se designan apropiadamente, pueden ser una herramienta poderosa y novedosa para obtener objetivos de conservación mientras se beneficia a la gente local.

## Introduction

A critical question for conservation policy is whether interventions incur net costs or provide net benefits to the local people who are the most directly affected. There is now widespread acceptance that conservation policies should, at the very least, do no harm, and where possible should contribute to poverty alleviation (CBD 2008). Protected areas (PAs) are one of the most widely adopted policies, currently covering >12% of the terrestrial land surface (UNEP-WCMC 2012). The debate around the impacts of PAs has, however, been particularly contentious. Large numbers of case studies document costs PAs have imposed on local people, such as restrictions on agriculture or access to natural resources (e.g., Brockington & Igoe 2006; Coad et al. 2008; Roe 2008). The concern about such negative impacts is a key reason why newer policies, such as payments for environmental services (PES), which provide benefits to local people conditional upon achieving an environmental outcome or a change in behavior, have gained popularity (Ferraro & Kiss 2002; Wunder 2007). It is hypothesized that PES improves local well-being due to the benefits provided (Ferraro & Kiss 2002; Pagiola et al. 2005). This hypothesis has, however, not been tested with empirical data (Pattanayak et al. 2010).

Rigorous impact evaluation methods are widely credited with having transformed development policy by quantifying the contribution that specific interventions have made to improvements in human well-being (Banerjee & Duflo 2011). There have been calls for adoption of the same methods in environmental science (Ferraro & Pattanayak 2006). Most published studies to date have focused on assessing environmental rather than social outcomes, for example, using impact evaluation methods to show that PAs and PES policies do indeed protect forests (Andam et al. 2008; Joppa & Pfaff 2011; Arriagada et al. 2012). These studies have also shown the critical importance of making comparisons with appropriate controls to avoid overestimating the effectiveness of interventions (Andam et al. 2008). Only 3 studies have evaluated the social impacts of PAs in developing countries, and in these cases PAs had no net impact or slightly positive impacts for local people (Andam et al. 2010; Sims 2010; Naughton-Treves et al. 2011). Our study is one of the first impact evaluations of the effects of PES on well-being in a developing country.

Published social impact evaluations also focus on net impacts determined via single poverty measures, whereas human well-being is complex and multifaceted (Scoones 1998; Agrawal & Redford 2006; McGregor 2007). Interventions with minimal effects on income may nonetheless contribute to less tangible aspects of well-being such as access to resources and education. There is also a need to disaggregate outcomes in order to understand the impacts of conservation interventions on different subsets of society (Daw et al. 2011), especially vulnerable groups. Impacts must also be understood in the context of a dynamic system, particularly as rural populations become increasingly linked to markets.

We used impact evaluation methods to quantify the impact of PAs and PES over time on a panel of intervention and matched control households practicing a range of livelihood strategies in villages in the northern forests of Cambodia. Northern Cambodia was an ideal location to test the impacts of PAs and PES because the interventions we studied were initiated relatively recently, thereby allowing before–after comparisons to be made, the PAs had existing residents, and the PES schemes have been well-documented (Clements et al. 2010). We considered whether PAs and PES delivered additional environmental outcomes relative to the counterfactual; the impact of PAs on multiple aspects of local well-being; the additional impact of PES programs on local well-being relative to the counterfactual; and the differential impacts of these interventions on different livelihood strategies.

## Methods

### Study Site

Our study site was the core management zones of 2 PAs in northern Cambodia (Supporting Information): Kulen Promtep Wildlife Sanctuary (gazetted 1993) and Preah Vihear Protected Forest (gazetted 2002). The PAs are in remote forest areas and contain 16 long-established villages. Local people were primarily subsistence farmers, practicing either rain-fed paddy rice or shifting cultivation and were dependent on forest resources both as a safety net and for cash income, particularly from the sale of liquid resins from dipterocarp trees (McKenney et al. 2004). Under Cambodian law, local uses of natural resources within PAs are legal, although forest clearance, commercial logging, and hunting or trade in threatened species are illegal. Both PAs were paper parks until active management started in 2005. Villages were permitted by PA authorities to expand agriculture to a limited extent within agreed land-use plans. Three PES programs were instituted in villages within the PAs to complement PA management: direct payments for protection of nests of globally threatened birds in 6 villages; community-managed ecotourism conditional upon wildlife and habitat protection in 2 villages; and payment of premium prices for agricultural goods to households that kept to the land-use plans in 4 villages, which included those with ecotourism and the Bird Nests protection program (Ibis Rice) (Clements et al. 2010).

### Survey Design

Impact evaluation methods (quasi-experimental matching and difference-in-difference) were used to control for observable and unobservable sources of bias in order to provide confidence that observed differences were due to the PA and PES interventions rather than other factors. Quasi-experimental matching was used to control for possible observable sources of bias by selecting control groups that were as similar as possible to the treatment groups prior to the initiation of the interventions. Matching assumes that there were no sources of unobserved bias. Difference-in-difference estimators control for time-invariant unobservable characteristics through the use of data from the same treatment units over time (Wooldridge 2002). A key assumption is that the expected trend in the outcome variable for the control group is equal to the expected trend for the intervention group, in the absence of the intervention. Combining the methods, by using matching to select the control groups, can help reduce this source of bias; this is called a BACI study (before–after, control intervention).

Environmental outcomes were measured using deforestation rates in 1-km grid squares because the PAs and 2 of the PES schemes explicitly targeted forest protection. Social outcomes were based on assessed household well-being. For the assessment of PA impacts, we used 3 comparison groups: villages within PAs; control villages >20 km from the PA boundaries that were matched to the within-PA group based on observed variables in 2005, prior to initiation of the interventions; and villages bordering the PAs (4–12 km from the PA boundary) because studies usually assess PA impacts in comparison with nearby areas (Joppa & Pfaff 2010). Environmental outcomes were assessed using a full BACI study comparing the deforestation rates around within-PA villages before and after PA management started with the matched control group. Social outcomes of PAs could not be assessed using a full BACI study because data on household well-being was not available prior to the start of PA management. Instead, outcomes were assessed in comparison with the matched control villages only, following the same panel of households over time.

Environmental impacts of PES were assessed using a full BACI study that compared the deforestation rate in grid squares around villages receiving forest protection payments (ecotourism and Ibis Rice) with matched control squares around other villages within the PAs that did not receive payments, both before and after payments were initiated. Social impacts of PES were assessed using difference-in-difference estimators that compared households that received payments with those in the same villages that did not, before and after the initiation of payments.

### Selection of Matched Control Villages for within-PA Villages

For the PA impact assessment, potential matches to the 16 within-PA villages were chosen from a database of all 211 villages in Preah Vihear province. The matching variables selected were forest cover within 5 km of the village, village size, and distances to roads and markets, all in 2005. These variables were the main factors thought to have influenced PA placement and were the main determinants of poverty at the village level prior to the initiation of the PAs (Supporting Information). We carried out nearest-neighbor covariate matching using the Mahalanobis distance (Abadie & Imbens 2006) in R version 2.14.2 to ensure that the sample of intervention and control villages were not significantly different (Supporting Information). We then used random stratified sampling by district to select 7 control villages that were >20 km from the PAs and distributed across the landscape. Matching ensured that the control villages were as similar as possible to the within-PA villages for observed baseline characteristics in 2005 (prior to the initiation of the interventions) that would be expected to have significant effects on local livelihoods and deforestation rates.

### Environmental Outcome Measure

Deforestation rates were calculated between the 2001 to 2002 dry season (hereafter 2001/2002) to the 2005 to 2006 dry season (hereafter 2005/6), the 4 years immediately prior to establishment of the PAs, and from 2005/6 to the 2009 to 2010 dry season (hereafter 2009/10), when the PAs were actively managed and PES schemes were operational. We used all 16 villages within the PAs (4 of which had received Ibis Rice and ecotourism payments), the 7 controls, and all 11 villages that bordered the PAs. Forest and non-forest areas were mapped for all 1-km grid squares within 8 km of the villages at a resolution of 1 ha (Supporting Information). We chose the 8 km radius because this was the maximum distance local people traveled to agricultural plots. For the analysis of PA impacts, grid squares were assigned a treatment type depending on whether they were within a PA (*n* = 1356), around a control village (*n* = 913), or bordering a PA (*n* = 1035). For the analysis of PES impacts within the PAs, squares were assigned a treatment type depending upon whether they were within 5 km of one of the 4 PES villages (*n* = 217) or were within 5 km of another village in the PA that was not receiving payments (*n* = 433). We used the reduced radius of 5 km to separate effects due to the different villages because the minimum distance between villages was 10 km. A second level of matching was used to compare the effects of the treatments on similar 1-km grid squares. The base forest area in 2001/2002 (for the first 4-year period) or 2005/2006 (for the second 4-year period), the distance to nearest village, and slope were used as the matching variables because these variables are known to affect deforestation rates. The variance of the matching estimator was adjusted to account for the clustering of grid squares around villages (Hanson & Sunderam 2012). Covariate balancing tests confirmed that the 2 selected samples were closely matched (Supporting Information).

### Social Outcome Measures

We used 4 measures of household well-being: poverty, determined with the Basic Necessities Survey (Davies 2006; Supporting Information); rice harvests, because rice is the staple crop in Cambodian diets; food security; and education level of each household member. Households were categorized by which livelihood strategies they practiced (e.g., resin tappers, shifting cultivators). Livelihood data were collected in 2008, 3 years after PA management was initiated and before households were paid from any of the PES programs, and in 2011, after households had been receiving payments for 1–3 years (depending on the program and the household). Surveys were conducted by trained Cambodian social researchers in 20 villages: 11 within the PAs, 5 matched controls, and 4 villages bordering the PAs (Supporting Information). The first assessment of 871 households was conducted in September–November 2008, 3 years after the PA management activities were initiated and before the market-linked PES programs were scaled up. Households were selected through random stratified sampling based on a participatory wealth ranking exercise in each village. The second assessment took place in July–September 2011 for the same households and an expanded sample (1053 total). Twelve percent of the original sample could not be located, either because people had moved away or were absent. We interviewed 769 households in both periods (443 within PAs, 185 controls, and 141 in border villages). In the PA impact evaluation, we used the entire panel of 769 households. In the PES impact evaluations, we used a subset of villages from within the PAs: 174 households from 4 villages for ecotourism and Ibis Rice, of which 27 and 50 households respectively were paid during 2008–2011; and 247 households from 6 villages for the bird nest protection payments, of which 28 were paid during 2008 to 2011.

Mixed effects models in R version 2.14.2 were used to analyze the factors influencing the well-being variables (poverty, rice harvests, and food security); village was included as a random effect (Pinheiro et al. 2011). Models were formulated for each variable in 2008 and 2011 and for the change in each variable between 2008 and 2011. Competing models were developed based on the main research questions and compared using second-order Akaike information criterion corrected (AICc) values calculated using maximum likelihood, removing terms with ΔAICc values of >4 (Burnham & Anderson 2002; Supporting Information). Final model coefficients were then estimated using restricted maximum likelihood (Burnham & Anderson 2002). Contrasts tests were used to compare households within PAs with households in control villages and with households on the border of PAs. Binomial categorical variables (e.g., if a household had a resin tapper) were analyzed using mixed effects models with a binomial error distribution. Models were used to compare the differences between interventions and years in terms of the livelihood strategies practiced by households and to determine which variables were characteristics of households that chose to sign up for PES programs. Education was expressed as whether a child was attending high school or not.

## Results

### Additional Impacts of PAs and PES on Environmental Outcomes

Deforestation rates within the PAs significantly decreased after PA management started in 2005/6 (*P* < 0.05), whereas deforestation rates increased significantly both in control areas and in the border areas around the PAs during the same period (Table1). Based upon the matching estimators, deforestation rates within the PAs were significantly less than in control areas after PA establishment, whereas there was no significant difference before PA establishment (Supporting Information). PAs reduced the deforestation rate by >60% in comparison with the control areas. The deforestation rate in border areas to the PAs was much greater than inside the PAs and increased significantly after the PAs were established (Table1). Border-PA villages were closer to markets and roads (Clements et al. 2014), which might be expected to increase deforestation rates, and there may also have been spillover effects from the PAs. Comparing border areas with PAs is therefore not an appropriate way to assess PA impacts (Joppa & Pfaff 2010). The PES interventions reduced deforestation rates within the PAs by an additional 50% over the 2005/2006 to 2009/2010 period (Table1). We have shown separately that the bird nests PES intervention was highly successful at protecting globally threatened birds (Clements et al. 2013).

**Table 1:** Changes in deforestation rates between the 4 years prior to establishment of the protected areas (PAs; 2001/2002 dry season to 2005/2006 dry season) and the subsequent 4 years after establishment (2005/2006 dry season to 2009/2010 dry season) for protected areas, matched control areas, and areas bordering the PAs, and for villages receiving payments (PES), and villages not receiving payments within the PAs

	*Landscape-level interventions*			*Within PAs only*	
	*PAs*	*Controls*	*Border areas*	*PES*	*No PES*
No. of 1-km grid squares (no. of villages)	1356 (16)	913 (7)	1035 (11)	217 (4)	433 (11)
Deforestation rate between 2001/2002 and	−0.872 (0.105)	−1.398 (0.173)	−2.193 (0.167)	−2.529 (0.477)	−0.534 (0.086)
2005/2006 before PA establishment (SE)^a^					
Deforestation rate between 2005/2006 and	−0.636 (0.058)	−2.001 (0.214)	−3.595 (0.194)	−0.734 (0.096)	−1.298 (0.151)
2009/2010 after PA establishment (SE)^a^					
Difference between periods^b^	0.236*	−0.603*	−1.402***	1.795***	−0.765***
Matching estimator clustered by village^b^,^c^		−1.152*	−2.352***		−0.712*
Effect size		3.947	3.801		2.144

a Data are based upon the average deforestation rate (in hectares) of 1-km grid squares in the areas surrounding the villages.

b Significance:

* *P*< 0.05;

**P < 0.01;

*** *P* < 0.001.

c The matching estimator indicates the significance of the difference between the deforestation rate within PAs and controls or border areas and between the deforestation rate around villages within PAs receiving payments and villages not receiving payments. Hence, it gives the estimated effect of interventions from 2005/2006 (when implementation started) until 2009/2010.

### Changes in Livelihoods Over Time

National economic growth in Cambodia averaged nearly 10% from 1998 to 2008, declined to 0.1% in 2009 during the global financial crisis, and recovered to 6–7% from 2010 to 2012 (World Bank 2012). In the context of this rapid economic development, it is unsurprising that the well-being of households in the panel increased significantly between 2008 and 2011 (Table2) and that this increase was seen both for the poorest and richest quintile in the sample (Supporting Information). On average, household poverty decreased, agricultural productivity and food security improved, land holdings increased, and more households operated family businesses and adopted mechanized agriculture. Households switched from shifting cultivation—which requires lower inputs but is less productive—to paddy rice and began growing cash crops. Increased mechanization was funded by sales of assets, particularly resin and livestock, and led to declines in the number of cattle and the use of animals for agriculture (Table2 & Supporting Information). Households that were poorer in 2008 were less likely to make these switches and tended to be less educated, had fewer working adults, younger household heads, and fewer assets and were unlikely to operate family businesses or have jobs (Supporting Information).

**Table 2:** Household well-being and livelihood strategies for a panel of 769 households bordering protected areas (PAs), within PAs, and in matched control areas outside PAs in northern Cambodia, 2008–2011

							*Test of difference*^a^		
	*Border PA*		*Within PA*		*Control*		*(within-PA vs. controls)*		
	*2008*	*2011*	*2008*	*2011*	*2008*	*2011*	*2008*	*Change*	*2011*
Households	141	141	443	443	185	185			
Well-being variables									
Poverty	10.5	12.5	9.6	11.8	8.0	11.4	*^b^	ns	ns
Rice harvest (kg)	2181	3015	1851	2506	1293	2329	ns	ns	ns
Food security (kg)	219	1942	−230	1337	−633	1109	ns	ns	ns
Livelihood strategies^c^									
Resin tapper (%)	32	30	55	59	28	37	***	ns	***
Rice farmer (%)	94	96	91	96	94	95	ns	ns	ns
>1 ha of paddy fields (%)	90	90	73	85	63	79	*	ns	ns
Mini tractor (%)	36	54	30	60	26	37	ns	**	***
Rice shifting cultivation (%)	38	27	37	26	45	39	*	*	**
Cash crops	n/a	5	n/a	2	n/a	10			**
Employed (%)	11	10	6	9	3	4	ns	ns	*
Service or shop (%)	23	24	14	26	14	29	ns	ns	ns

a Tests of difference are mixed effects regression models for continuous variables (poverty, rice harvest, food security, cattle) and generalized mixed effects models with a binomial link function for categorical variables.

b Tests of difference significance values: ns, not significant;

* *P* < 0.05;

** *P* < 0.01;

*** *P* < 0.001.

c Households could have more than one livelihood strategy.

### Additional Impacts of PAs on Well-Being

In both 2008 and 2011, households bordering PAs were less poor, had larger rice harvests, and were more food secure than households within PAs or controls (Table2 & Supporting Information). Households bordering PAs also increased their rice harvests at a greater rate than the other treatment groups (Supporting Information). Border households were better off because they were closer to roads, markets, and services than both households within-PAs and controls (Clements et al. 2014), demonstrating that comparing within-PA groups with nearby villages is not a valid way to assess PA impacts (Joppa & Pfaff 2010).

PAs had limited impact on household poverty relative to matched controls. The within-PA group was significantly better off than the control group in 2008 (Table2 & Supporting Information), and there was no significant difference between the 2 groups in both the rate of change in household poverty status and household poverty status in 2011. For the other main well-being variables, rice harvest and food security, there were no significant differences in either the absolute values or the rate of change (Supporting Information). The percentage of residual variance explained by the village term was low in all models, implying that unobserved factors at the village level did not bias the results (Supporting Information). The overall impact of PAs on households was therefore quite limited, which suggests that rates of change were mainly due to larger economic factors at the landscape or national level, such as Cambodia's rate of economic growth during the study period.

### Differences in PA Impacts among Livelihood Strategies

There was considerable similarity in the prevalence of some livelihood strategies between within-PA and control households in 2008; however, households within PAs were more reliant on nontimber forest products, particularly resin, than control households (Table2). Control households were more likely to practice shifting cultivation for rice, and this difference between the controls and within-PA households significantly increased from 2008 to 2011. Control households were also far more likely to be growing cash crops in 2011. The PA authorities strictly restricted expansion of shifting cultivation and cash crops, which probably explains these differences. Employment rates increased within PAs during 2008–2011, in comparison with controls, principally due to hiring of local villagers by the PAs.

**Figure 1:**
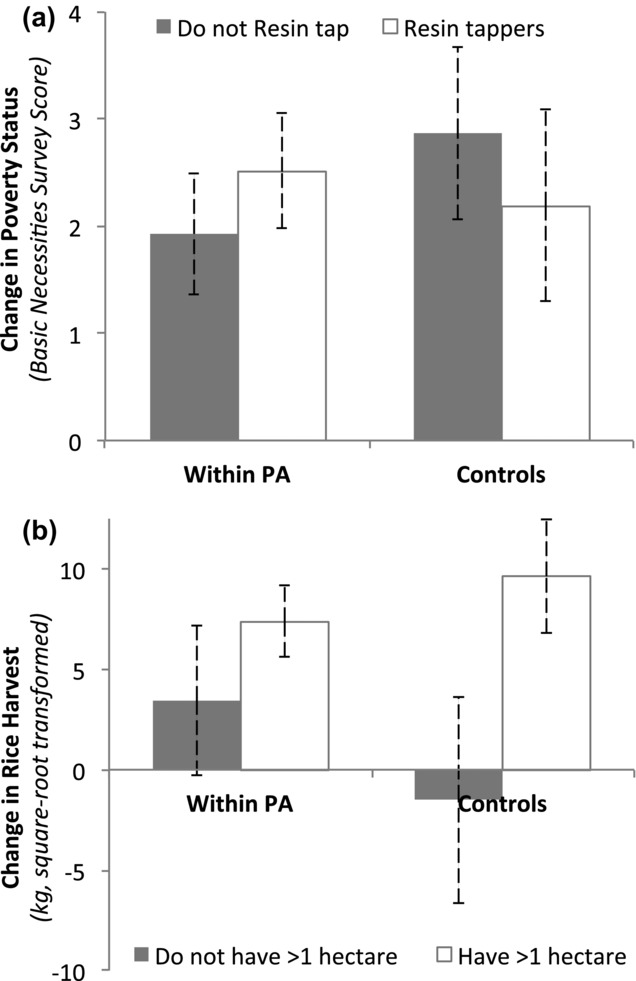
Effect of protected areas on (a) poverty status of resin tappers, measured using the Basic Necessities Survey score, and (b) rice harvests of households with >1 ha of paddy. The graphs show the predicted effects and 95% confidence intervals from the mixed effects model (within protected area [PA] *n* = 443 households; control areas *n* = 185 households).

Although the average rates of change in poverty status were similar among households within PAs and controls, significant differences were observed for different livelihood strategies. Within PAs, resin tappers improved their economic status at a greater rate than those who did not tap resin, whereas the reverse was observed for the controls (Fig.1a & Supporting Information & significance *P* < 0.01). Poverty reduction rates for those who did not tap resin were therefore significantly slower within PAs than outside PAs, perhaps because control households could practice other forms of agriculture (shifting cultivation and cash crops) that were restricted by the PA authorities. Tenure security over resin trees was greater within PAs, potentially explaining why resin tappers within PAs, who made up 59% of within-PA households in 2011 (Table2), did significantly better.

Land tenure security was also higher within PAs, whereas some control households lost access to land for farming due to development pressures. Consequently rice harvests of control households that did not own >1 ha of paddy fields declined slightly, whereas similar households within PAs showed continued improvements in rice harvests (Fig.1b & Supporting Information & significance *P* < 0.05). Households that did not own >1 ha made up a minority of households under each treatment type: 21% of households outside PAs and 15% of households within PAs (Table2).

### Participation in PES Programs

Entry into 2 of the 3 payment programs was not random. Households that decided to sign up for the Ibis Rice and ecotourism programs had greater rice harvests, were more food secure, and were more likely to have >1 ha of paddy fields than households that chose not to participate in 2008 (Table3). Ibis Rice households were also less poor and more likely to use machinery. In the case of Ibis Rice, this is understandable because only net rice producers could afford to sell excess rice to the program. The ecotourism program required households to divert labor from agriculture to invest in tourism activities, which again suggested that households that were more food secure were more likely to sign up. As a consequence participants in the ecotourism program were also more likely to participate in the Ibis Rice program (Fisher's exact test of independence, *P* = 0.06). For the ecotourism program, some elite capture may have occurred; participants in 2008 were more likely to be employed, particularly in the public sector (teachers, village chiefs, and commune councilors), although by 2011 they had mostly been replaced. The ecotourism program also positively targeted poor female-headed households through participation in a women's group that sold supplies to tourists; all Ibis Rice households were headed by men. Households engaged in the Bird Nests program were similar to other households in the village, probably because households received immediate payments to cover the costs of nest protection (and then a bonus if the nests were successful), so there were fewer barriers to entry. Engagement in the Bird Nests program was independent of participation in the other 2 programs (Fisher's exact test, *P* = 1 and *P* = 1).

**Table 3:** Differences between household status and livelihood strategies in 2008 before the commencement of the payments for environmental services (PES) programs for households that participated in the PES programs and households that did not.^a^

	*Bird nest participants*^b^			*Ecotourism participants*^c^			*Ibis Rice participants*^c^		
	*Yes*	*No*	*P*^d^	*Yes*	*No*	*P*^d^	*Yes*	*No*	*P*^d^
No. of households	28	219		27	147		50	124	
Female-headed households (%)	7	5		11	5		0	8	
Well-being variables									
Poverty	9.4	9.4	ns	10.8	10.2	ns	11.1	9.9	*
Rice harvest (kg)	2154	1935	ns	2811	1926	**	2707	1804	***
Food security (kg)	−194	−154	ns	304	−191	(*)	486	−357	**
Livelihood strategies									
Resin tappers (%)	64	55	ns	56	56	ns	54	57	ns
Rice farmer (%)	89	90	ns	93	88	ns	96	85	(*)
>1 ha of paddy fields (%)	68	72	ns	93	76	*	94	72	**
Mini tractor (%)	29	26	ns	33	25	ns	44	19	**
Rice shifting cultivation (%)	43	31	ns	11	18	ns	10	19	ns
Employed (%)	0	9	ns	19	7	(*)	14	6	ns
Service or shop (%)	14	12	ns	19	13	ns	14	14	ns
Average annual payments per household,		132	(18)		225	(14)		413	(41)
US$ (SE)									
Percentage of households in the village		7	(616)		12	(499)		24	(616)
engaged in program (total households)									
Percentage of households engaged		10			62			54	
for > 1 year									

a Data are from the same villages within the protected areas.

b Data for the Bird Nests program are based on 6 villages (247 households). The Bird Nests program provided direct payments for protection of nests of globally threatened birds.

c Data for the Ecotourism and Ibis Rice programs are based on 4 villages (174 households). The ecotourism program provided payments conditional on wildlife and habitat protection, and Ibis Rice provided households with premium prices for agricultural goods if they kept to agreed land-use plans.

d Tests of difference are mixed effects models with a binomial link function. Significance: ns, not significant;

* *P* < 0.1; *P < 0.05;

** *P* < 0.01;

*** *P*< 0.001.

### Additional Impacts of PES Programs on Well-Being

Households that signed up to the Ibis Rice and ecotourism programs improved their poverty status at a greater rate than non-PES households from the same villages, even when we accounted for the fact that some started at a higher baseline level in 2008 (Table4; *P* < 0.05 in both cases). Ibis Rice households also increased their rice harvests and improved their food security at a faster rate than other comparable households (Table4; *P* < 0.01 in both cases). Households that received high payments from the ecotourism and Ibis Rice programs were able to afford to keep their children in school for longer and to pay for them to attend secondary and high schools away from their home villages (Table4; *P* < 0.01). The Bird Nests program had no additional impact on household well-being, perhaps because the payments were significantly lower than the other schemes (Table4). The models for the PA impact evaluation also showed that the Ibis Rice and ecotourism programs improved household well-being (Supporting Information).

**Table 4:** Effects of the payment for environmental service (PES) programs on the change in 4 measures of well-being between 2008 (before payments) and 2011 (after payments).

	*Poverty*^a^,^b^		*Rice harvest*^b^		*Food security*^b^		*Education*^b^,^c^	
Intercept	5.627	***	26.020	***	60.507	***	−3.122	ns
Base variable	−0.386	***	−0.503	***	−0.746	***		
Ibis Rice program, payment	0.058	***	0.381	***	0.297	***	0.110	*
Ecotourism program, payment	0.053	*	0.003	ns	−0.029	ns	0.074	(*)
Bird Nests program, payment	−0.022	ns	−0.053	ns	0.078	ns	−2.048	ns
Household head education level							−0.739	ns
Change in poverty							0.311	ns
Random effect of households: Percentage residual variation							0.0	
Random effect of village: Percentage residual variation	9.3		6.4		5.6		29.5	

a Mixed effects models (poverty, rice, food security) are based on a panel of 174 households from 4 villages where the PES schemes were in operation. Fifty households were involved in the Ibis Rice scheme, 27 were involved in ecotourism, and 16 received direct payments for protection of bird nests.

b Significance: ns, not significant;

* *P* < 0.1; *P < 0.05;

** *P* < 0.01;

*** *P* < 0.001.

c Education is represented by whether a child was attending a high school (in a district or provincial town). The education model had a binomial link function based on a panel of 36 children who had completed primary school by 2008 (28 households out of the 174).

## Discussion

The importance of ecosystem services to overall human well-being is well recognized (MEA 2005), but interventions to manage and conserve ecosystem services may impose costs (Brockington & Igoe 2006; Cernea & Schmidt-Soltau 2006; Coad et al. 2008) as well as benefits (Wunder 2001; Scherl et al. 2004; Coad et al. 2008) on local people. We used rigorous impact evaluation methods to analyze both the social and environmental impacts of interventions to conserve and maintain ecosystem services, through PAs and PES.

We found that since their initiation, PAs and PES have delivered additional conservation outcomes in northern Cambodia, relative to the counterfactual, in terms of protection of tropical forests from deforestation and, for at least one of the PES programs, protection of globally threatened wildlife species (Clements et al. 2013). The principal effect of the PAs since the start of active management was to mitigate external drivers of ecosystem loss (especially deforestation), particularly in-migration to existing villages, formation of new settlements, and the gazettement of large-scale concessions for agro-industrial development within PAs (which began after 2008). Implementation of the PES programs would have been impossible without this protective effect of the PAs; the 2 conservation strategies are complementary.

As a consequence of the exclusion of outsiders from the PAs, local people have been able to continue to use forest and land resources for their livelihoods based upon their legal rights under Cambodian law, including use of forest resources (especially resin) and farming within agreed land-use plans. No resettlement occurred. The Cambodian PAs are therefore very different from strictly enforced PAs, but they share many characteristics with the estimated 56–85% of PAs in developing countries that contain local people (Brockington & Igoe 2006). Our principal finding was that under these conditions PA management had minimal impacts on the livelihoods of local residents on average, which is consistent with the PAs being primarily designed to protect ecosystems from external drivers of loss. Instead, the improvements in well-being seen across all treatment groups were driven largely by Cambodia's rapid economic growth. PA management restricted livelihood opportunities for local people by limiting crop types (principally shifting cultivation and cash crops) and some land clearance. Conversely, PAs provided notable benefits for forest resource users (such as resin tappers), who gained from the restrictions on outsiders (Clements et al. 2014). Continued and unsustainable use of natural resources in PAs by local residents can, however, lead to trade-offs from a biodiversity conservation perspective, as such PAs may be less effective at conserving key species (O'Kelly et al. 2012).

Ours is one of the first studies to evaluate the social impacts of PES programs. Our results provide empirical support for suggestions that the impacts of PES on human well-being depend fundamentally upon program design (Pagiola et al. 2005; Wunder 2008). PES can support social goals by minimizing constraints on program entry by the poor and providing sufficient incentives to offset the opportunity costs of participation and thereby increasing overall human well-being. The PES program entry constraints might include eligibility requirements or abilities, which the poor would be less likely to fulfill (Wunder 2008). Of the 3 PES programs we evaluated, the Ibis Rice program had the most significant entry constraints; participants needed to have sufficient land to produce an agricultural surplus to sell to the program. By contrast, the Bird Nests program, which provided direct cash payments for protection of biodiversity, required no capital assets to join and provided a portion of the payment up-front, allowing any household to participate. The pro-poor impacts of ecotourism are known to be limited by the additional capabilities required to engage in tourism (Kiss 2004). The Cambodian ecotourism program specifically contained pro-poor provisions, which mitigated these barriers to some extent.

There was no evidence that any of the PES programs led to net negative impacts on local well-being, and the 2 market-based programs (Ibis Rice and the Ecotourism) had significant net positive impacts for their participants. The development benefits of the 3 programs are linked to the magnitude of the payments made. Under the Bird Nests program, payments were low, based upon the minimum daily wage local people were willing to accept (the opportunity cost of a day's work). Despite the lack of constraints on access, suggesting it should be the most pro-poor of the interventions, the additional livelihood benefit of the program was limited. By contrast, payment levels under the 2 market-based programs were based on the market's willingness to pay for the additional environmental outcomes, which was high.

The robustness of these results depends upon the extent to which the survey design adequately addressed potential sources of bias. The deforestation results were based on a full BACI survey and hence are likely to be the most robust. The key assumption was that protected forest areas were similar to unprotected forest control areas, which is likely given that the placement of PAs was based primarily upon remoteness, rather than differences in productivity, and because the landscape is relatively uniform and there were suitable large, remote, forested areas that remained unprotected. The intervention and control areas for both the PA and PES analyses had similar deforestation rates prior to the initiation of the conservation programs in 2005, which supports this assumption.

The assessment of the social impact of PAs was based on the matching of the within-PA and control villages and assumed that households within the 2 groups practiced similar livelihood strategies when PA management started in 2005 and that the observed subsequent divergence in poverty status and livelihood strategies was due to the interventions rather than other factors. These assumptions are reasonable because 2008 livelihood assessments suggested that the within-PA and control villages were very similar and differed little from other remote forest villages in the same landscape in the mid-2000s (McKenney et al. 2004). Over the study period other factors that would affect poverty (such as development interventions) tended to be implemented relatively uniformly over the province. The analysis of subgroup effects, for resin tappers and landowners, was based upon an a priori hypothesis that these groups are affected by development pressures outside PAs (Clements et al. 2014). We assumed that membership of these groups, with respect to the interventions, was random, which is reasonable given that a large number of people across the landscape practice these livelihood strategies. The PES social impact assessment was based only on a difference-in-differences estimator and assumed that the trend of payment and non-payment households would have been similar in the absence of the payments and that selection was not contingent upon time-lagged variables. This is reasonable given that both groups of households were selected from the same villages and the major factors affecting poverty status (market access, agricultural productivity, development projects) would exert their influence across the entire village.

The combination of the PA and PES interventions described here delivered additional environmental outcomes, relative to the counterfactual, and had 3 important social impacts: securing forest resources for local residents, which benefited some groups whilst imposing costs on others; providing new significant sources of cash income for households that could afford to engage in the PES programs; and delivering net positive impacts for at least some of the PES participants. Our results confirm that PES, when designed appropriately, can be a powerful new tool for delivering conservation outcomes whilst benefiting local people, particularly as a complement to more traditional conservation interventions such as PAs.

## Supporting Information

Descriptions of the study sites and interventions (Appendices S1 and S2); survey design and impact evaluation methods, including the balancing statistics and tests for the covariate matching (Appendices S3-S7); methods used to assess deforestation rates (Appendices S8 and S9) and the deforestation analyses (Appendices S10 and S11); changes in household well-being (Appendices S12-S15); and the household well-being mixed effects models (Appendices S16-S22) are available online. The authors are solely responsible for the content and functionality of these materials. Queries (other than absence of the material) should be directed to the corresponding author.

## References

[b1] Abadie A, G Imbens (2006). Large sample properties of matching estimators for average treatment effects. Econometrica.

[b2] Agrawal A, K Redford (2006). Poverty, development, and biodiversity conservation: Shooting in the dark?.

[b3] Andam KS, PJ Ferraro, A Pfaff, GA Sanchez-Azofeifa, JA Robalino (2008). Measuring the effectiveness of protected area networks in reducing deforestation. Proceedings of the National Academy of Sciences.

[b4] Andam KS, PJ Ferraro, KR Sims, A Healy, MB Holland (2010). Protected areas reduced poverty in Costa Rica and Thailand. Proceedings of the National Academy of Sciences.

[b5] Arriagada RA, PJ Ferraro, EO Sills, SK Pattanayak, S Cordero-Sancho (2012). Do payments for environmental services affect forest cover? A farm-level evaluation from Costa Rica. Land Economics.

[b6] Banerjee A, E Duflo (2011). Poor economics: a radical rethinking of the way to fight global poverty.

[b7] Brockington D, J Igoe (2006). Eviction for conservation: a global overview. Conservation and Society.

[b8] Burnham KP, DR Anderson (2002). Model selection and multimodel inference: a practical information-theoretic approach.

[b9] CBD (Convention on Biological Diversity) (2008). Decision IX/18.

[b10] Cernea M, K Schmidt-Soltau (2006). Poverty risks and national parks: policy issues in conservation and resettlement. World Development.

[b12] Clements T, A John, K Nielsen, A Dara, T Setha, EJ Milner-Gulland (2010). Payments for biodiversity conservation in the context of weak institutions: comparison of three programs from Cambodia. Ecological Economics.

[b14] Clements TJ, H Rainey, D An, V Rours, S Tan, S Thong, WJ Sutherland, EJ Milner-Gulland (2013). An evaluation of the effectiveness of a direct payment for biodiversity conservation: the bird nest protection program in the northern plains of Cambodia. Biological Conservation.

[b13] Clements T, S Suon, D An, D Wilkie, EJ Milner-Gulland (2014). Impacts of protected areas on local livelihoods in Cambodia. World Development.

[b11] Coad L, A Campbell, L Miles, K Humphries (2008). The costs and benefits of protected areas for local livelihoods: a review of the current literature.

[b15] Davies R (2006). http://www.mande.co.uk/special-issues/the-basic-necessities-survey/.

[b16] Daw T, K Brown, S Rosendo, R Pomeroy (2011). Applying the ecosystem services concept to poverty alleviation: the need to disaggregate human well-being. Environmental Conservation.

[b17] Ferraro PJ, A Kiss (2002). Direct payments to conserve biodiversity. Science.

[b18] Ferraro PJ, SK Pattanayak (2006). Money for nothing? A call for empirical evaluation of biodiversity conservation investments. PLoS Biology.

[b19] Hanson SG, A Sunderam (2012). The variance of non-parametric treatment effect estimators in the presence of clustering. Review of Economics and Statistics.

[b20] Joppa LN, A Pfaff (2010). Reassessing the forest impacts of protection: the challenge of nonrandom location and a corrective method. Annals of the New York Academy of Sciences.

[b21] Joppa LN, A Pfaff (2011). Global protected area impacts. Proceedings of the Royal Society B.

[b22] Kiss A (2004). Is community-based ecotourism a good use of biodiversity conservation funds?. Trends in Ecology & Evolution.

[b23] McGregor JA, I Gough, JA McGregor (2007). Researching human well-being: from concepts to methodology. Well-being in developing countries: new approaches and research strategies.

[b24] McKenney B, C Yim, T Prom, TD Evans (2004). Focusing on Cambodia's high value forests: livelihoods and management.

[b25] MEA (Millennium Ecosystem Assessment) (2005). Ecosystems and human well-being.

[b26] Naughton-Treves L, J Alix-Garcia, CA Chapman (2011). Lessons about parks and poverty from a decade of forest loss and economic growth around Kibale National Park, Uganda. Proceedings of the National Academy of Sciences.

[b27] O'Kelly H (2012). Identifying conservation successes, failures and future opportunities; assessing recovery potential of wild ungulates and tigers in eastern Cambodia. PLoS One.

[b28] Pagiola S, A Arcenas, G Platais (2005). Can payments for environmental services help reduce poverty? An exploration of the issues and the evidence to date from Latin America. World Development.

[b29] Pattanayak SK, S Wunder, PJ Ferraro (2010). Show me the money: Do payments supply ecosystem services in developing countries?. Review of Environmental Economics and Policy.

[b30] Pinheiro J, Bates D, DebRoy S, Sarkar D, R Development Core Team (2011). http://cran.r-project.org/web/packages/nlme/index.html.

[b31] Roe D (2008). The origins and evolution of the conservation-poverty debate: a review of key literature, events and policy processes. Oryx.

[b32] Scherl LM, A Wilson, R Wild, JM Blcokhus, P Franks, JA McNeely, T McShane (2004). Can protected areas contribute to poverty reduction? Opportunities and limitations.

[b33] Scoones I (1998). Sustainable rural livelihoods: a framework for analysis.

[b34] Sims KRE (2010). Conservation and development: evidence from Thai protected areas. Journal of Environmental Economics and Management.

[b35] UNEP-WCMC (United Nations Environment Programme-World Conservation Monitoring Centre) (2012). The world database on protected areas.

[b36] Wooldridge JM (2002). Econometric analysis of cross section and panel data.

[b37] World Bank (2012). http://data.worldbank.org.

[b38] Wunder S (2001). Poverty alleviation and tropical forests—what scope for synergies?. World Development.

[b39] Wunder S (2007). The efficiency of payments for environmental services in tropical conservation. Conservation Biology.

[b40] Wunder S (2008). Payments for environmental services and the poor: concepts and preliminary evidence. Environment and Development Economics.

