# Supplementary material for: Impact of payments for environmental services and protected areas on local livelihoods and forest conservation in northern Cambodia
Source: Conserv Biol. 2014 Dec 9;29(1):78–87. doi: 10.1111/cobi.12423 (PMC4312980; doi:10.1111/cobi.12423)
Supplement: Supplementary file 1 — Descriptions of the study sites and interventions (Appendices S1 and S2); survey design and impact evaluation methods, including the balancing statistics and tests for the covariate matching (Appendices S3-S7); methods used to assess deforestation rates (Appendices S8 and S9) and the deforestation analyses (Appendices S10 and S11); changes in household well-being (Appendices S12-S15); and the household well-being mixed effects models (Appendices S16-S22) are available online. The authors are solely responsible for the content and functionality of these materials. Queries (other than absence of the material) should be directed to the corresponding author. [file cobi0029-0078-sd1.docx]

**Clements T, Milner-Gulland EJ.** The impact of Payments for Environmental Services and Protected Areas on local livelihoods and forest conservation in Northern Cambodia

**Supporting Information**

**S1. Study Sites and Interventions**

Cambodian Protected Area (PA) boundaries were drawn on old (1950s-1960s) maps in the 1990s and early 2000s, based primarily upon habitat types, historical records and very limited fieldwork, due to ongoing conflicts at that time. In general PAs are located in remote forested areas, where road access is poor and local poverty is higher than the national average (World Bank, 2009). Most PAs contain established villages since the location of settlements was not known when the PA boundaries were drawn. These villages were not resettled. The impact evaluation focused on the core Management Zones of two PAs in Preah Vihear province of northern Cambodia (Fig. S1): 1,811km^2^ of Kulen Promtep Wildlife Sanctuary (KPWS) and 1,776km^2^ of Preah Vihear Protected Forest (PVPF). KPWS was declared in 1993 as part of the Nature Protected Area network managed by the Ministry of Environment, and PVPF in 2002 as a Protected Forest managed by the Forestry Administration of the Ministry of Agriculture, Forestry and Fisheries. Sixteen villages were located inside KPWS and PVPF (Fig. S1), all of which had existed since at least the 1960s, although there was considerable disruption in the 1970-90s, due to the civil war and forced resettlement by the Khmer Rouge. Resettled people subsequently returned to their original villages from the 1990s onwards. Local people are primarily subsistence farmers, practicing either rain-fed paddy rice cultivation or shifting cultivation, and are dependent upon forest resources as a crucial safety net and for cash income (McKenney & Prom, 2002; McKenney et al., 2004). One of the most important source of cash income is the sale of liquid resins from dipterocarp trees, which makes up 16-23% of household income, with resin-tapping households earning $100-$340/year (Evans et al., 2002; McKenney et al., 2004). Cutting and clearance of resin trees by companies or loggers has been met with significant local protests in the past, due to the importance of resin income to local households (McKenney et al., 2004).

Gazettment of the PAs protected those areas to some extent from large-scale development (such as agro-industrial concessions) until 2008, when the new Protected Areas Law permitted concessions inside PAs (Cambodia Daily, 2011). Both PAs remained essentially paper parks until the start of a long-term PA management capacity-building program in 2005 titled “Establishing Conservation Areas through Landscape Management”, which was implemented by Government agencies and the Wildlife Conservation Society (WCS), and funded by the Global Environment Facility (GEF) through the United Nations Development Program (UNDP), with co-financing from WCS, the Critical Ecosystem Partnership Fund^[[1]](#footnote-1)^, the Danish International Cooperation Agency (Danida), the UK Department for International Development (DfID) and UNDP. As a consequence of the PA management capacity-building program, during 2005-2012 both PAs had funding of around $2-3/hectare for the core management zones, which is broadly comparable to PAs in developing countries (Bruner et al. 2004). PA authorities were charged with enforcement of Cambodian Law, under which local uses of natural resources are legal, although land clearance, cutting of timber for sale, and hunting or trade of threatened species are illegal. In-migration by outsiders to create new villages was prevented. The PA authorities were supported to undertake a participatory land-use planning process in each village to determine agreed land-use boundaries, within which villagers were permitted to expand their agriculture. The land-use plans provided the basis for the Ecotourism and Ibis Rice PES programs.

Three PES programs were designed to complement PA management (Clements et al. 2010). The programs were direct payments conditional upon protection of nests of globally threatened birds (Clements et al. 2012a), a community-managed ecotourism program that provided conditional support if villagers engaged in bird and habitat protection, and providing premium prices for agricultural goods if households limited field expansion to within agreed land-use plans (Ibis Rice; Clements et al. 2010). All three programs are classified as PES following Sommerville et al. (2009), who defined PES as approaches that aim to (*1*) transfer positive incentives to environmental service providers that are (*2*) conditional on the provision of the service, where successful implementation is based on a consideration of (*1*) additionality and (*2*) varying institutional contexts. The Bird Nests program started in 2003, and by 2008 was operating in six of the study villages (Clements et al. 2012a). The Ecotourism program was piloted in one village on a small-scale during 2005-2008, and was then scaled up in that village and expanded to cover a total of two villages during 2008-2011. The Ibis Rice program was initiated in 2008 in two villages and expanded to cover a total of four villages during 2008-2011, including those where the Ecotourism and the Bird Nests program were operating. The households analyzed in this study during 2008-2011 were new entrants into the PES programs and had not benefited previously from either the Bird Nests program or the piloting of the Ecotourism.

**Appendix S2.** Map of Preah Vihear province, Cambodia, showing the two Protected Areas (Kulen Promtep Wildlife Sanctuary and Preah Vihear Protected Forest) and their Management Zones, and the location of the three types of villages – those inside the Protected Areas, those bordering the Protected Areas and the Controls.

**S3. Impact Evaluation methods**

A fundamental goal of this study was to understand the impacts of the different conservation interventions – both PAs and the three PES programmes – on key environmental and social outcomes. Understanding impacts requires moving beyond simplistic analyses based upon correlations between an intervention and changes in an outcome variable, in order to be able to attribute impacts. Rigorous impact evaluation survey designs can be used to untangle the impacts of forest conservation policies from the wider dynamics of the system, by assessing the degree to which changes in outcome variables can be attributed to policy interventions as opposed to other factors (Ferraro, 2009). Typically this is achieved by evaluating outcomes in comparison with the counterfactual – what would have happened in the absence of the intervention. Standard approaches use randomized control trials with policy interventions assigned randomly to intervention and control sites in order to eliminate other sources of bias. In econometrics the difference between the intervention and the control groups is called the *average treatment effect*.

However placement of forest conservation interventions, such as protected areas (Joppa & Pfaff, 2010b), is usually non-random with respect to other areas in the landscape. In these cases, quasi-experimental survey designs such as matching can be used to control for other sources of bias by ensuring that intervention and control areas or groups are comparable in all aspects except that the controls have not received the intervention (Rosenbaum & Rubin 1983; Ferraro & Pattanayak, 2006; Ravallion, 2006; Pattanayak, 2009). Matching minimizes differences in observed covariates between intervention and control groups. The comparison between the intervention and control groups is therefore equivalent to what would have happened to the intervention groups had they not been subject to the intervention. In econometrics this is called the *average treatment effect on the treated*.

For matching to be applied properly, the underlying dynamics of the system being investigated must be well understood in order to select appropriate covariates (Ravallion, 2006). The matching covariates should not be affected by the interventions (i.e. they are exogenous to the system that is being studied). A critical assumption is that for the matching variables, potential outcomes are independent of assignment to control or intervention groups, called conditional independence. A second critical assumption is to ensure covariate balance (i.e. covariates have similar values for controls and intervention groups) and to ensure that the area of common support (covariate values found in both control and interventions groups) is large. A key problem is that matching can only be done on observables (i.e. observed covariates), leaving open the possibility of sources of unobserved bias. If an unobserved covariate does affect outcomes but was not controlled for by the matching process, then a difference between the intervention and control groups would be found when in fact the intervention had no effect.

Another method that can be used to reduce unobserved sources of bias is to follow trends in time for intervention and control groups, and to use this data to calculate the difference-in-difference estimator. The difference-in-difference estimator controls for time-invariant unobservable characteristics by using data from the same treatment units over time (Wooldridge, 2002). A key assumption is that the expected trend in the outcome variable for the control group is equal to the expected trend for the intervention group, in the absence of the conservation intervention. Combining the difference-in-difference method with matching, by using matching to select the control groups, can ensure that this assumption is met (this also called a before-after control-intervention study). Smith and Todd (2005) found that the difference-in-difference matching estimator performs best among other matching estimators, and Imbens and Woolridge (2009) recommend combining methods in this way. At least one other recent study (Arriagada et al. 2012) combined both matching and difference-in-difference estimators to evaluate the environmental impact of PES programmes. The same approach was used in this study, to calculate the impact of protected area and PES programmes in the Northern Plains of Cambodia.

A second methodological issue considered in the survey design was the scale at which interventions were implemented and outcome variables could be measured. Impacts were deliberately investigated at the local scale, in order to avoid the potential biases associated with large-scale studies (Arriagada et al. 2012). The primary unit that was affected by both the PA and PES programmes was the village, because this was the scale at which local people experienced the conservation interventions and responded to them. It was assumed that the different villages could be considered as independent replicates, because local people were generally sedentary farmers that used forest resources within a day’s walk (5-8 km) from their village. Within each village, different households would respond to the conservation interventions in different ways depending upon their livelihood strategies and attitudes and the extent to which they benefited from or were restricted by the conservation interventions. Consequently, a nested survey design was used: sampling independent replicates at the village-level, and then sampling different types of households within each village or 1 km grid squares around a village.

**S4. Survey Design**

Environmental outcomes were measured using deforestation rates in 1 km grid squares because the PAs and two of the PES schemes explicitly targeted forest protection. Social outcomes were based upon assessed household wellbeing. The assessment of PA impacts compared villages within PAs (the ‘within-PA group’) to matched control villages >20 km from the PA boundaries. The control villages were deliberately selected far from the PAs in order to reduce spillover effects, and were selected using baseline variables in 2005, which was the year PA management was initiated. The within-PA group was also compared to a group of villages bordering the PAs (4-12 km from the PA boundary), since studies have normally assessed PA impacts in comparison with nearby areas (Joppa & Pfaff 2010b). Two analyses were done:

1. Environmental impacts. This used a full BACI (before-after control-intervention) survey design, by following the deforestation trends around the within-PA and matched control villages before (2001 to 2002 dry season [2001/2] to 2005 to 2006 dry season [2005/6]) and after (2005/6 to 2009 to 2010 dry season [2009/10]) PA management started. Two levels of matching were done: firstly at the village scale (matching villages within PAs with potential controls >20km from the PA boundary) and then matching the 1 km grid squares around the two village groups. This ensured that the final matched sample was as similar as possible. Clustered matching estimators were used to account for the pseudo-replication, since there were multiple 1 km grid squares around each village. The key assumption is that protected forest areas were similar to unprotected forest control areas, which is likely given that the placement of PAs was based primarily upon remoteness, rather than differences in productivity, and because the landscape is relatively uniform and there were suitable large, remote, forested areas that remained unprotected. Both the within-PA and the matched control groups had very similar deforestation rates in the first time period (prior to PA management starting, see Table S4). This suggests that its plausible to assume that the trend in the outcome variable for the control group would be equal to the expected trend for the intervention group, in the absence of the intervention.

2. Social impacts. A full BACI study was not possible, because data on household wellbeing was not available prior to the start of PA management. Therefore instead outcomes were assessed in comparison with the matched control villages only, following the same panel of households over the period 2008 to 2011 (starting three years after PA management started and continuing to six years after PA management started). This approach is is less robust than a full BACI study and assumes that households within the two groups practiced similar livelihood strategies when PA management started in 2005, and that the observed subsequent divergence in poverty status and livelihood strategies was due to the interventions rather than other factors. These assumptions are reasonable because the 2008 livelihood assessments suggest that the within-PA and control villages were very similar, and were little different from other remote forest villages in the same landscape in the mid-2000s (McKenney et al. 2004). Over the study period other factors that would affect poverty (such as development interventions) tended to be implemented relatively uniformly over the province.

The assessment of PES impacts compared villages within PAs that received payments to villages inside PAs that did not receive payments. Two analyses were done:

1. Environmental impacts. This used a full BACI (before-after control-intervention) survey design, by following the deforestation trends around the PES and matched no-PES grid squares before (2001/2 to 2005/6) and after (2005/6 to 2009/10) payments management started. Both the PES and no-PES groups had very similar deforestation rates in the first time period (prior to payments starting, see Table S4). This suggests that its plausible to assume that the trend in the outcome variable for the control group would be equal to the expected trend for the intervention group, in the absence of the intervention (which is a key assumption of a difference-in-differences estimator).

2. Social impacts. A full BACI study was not possible, because although data on household wellbeing were available from 2008 (prior to payments), it was not possible to predict in 2008 which households would sign up for payments and which ones would not. Consequently it wasn’t possible to conduct a matching at the household level in the baseline year, and anyway this would have required data on every household in the village (in order to select the matches), which would have been logistically impossible to collect. Outcomes were assessed using difference-in-difference estimators only, following the same panel of households from the same villages over the period 2008 to 2011 (during which period payments were made). The analysis assumes that the trend of payment and non-payment households would have been similar in the absence of the payments. This is reasonable given that both groups of households were selected from the same villages, and the major factors affecting poverty status (market access, agricultural productivity, development projects) would exert their influence across the entire village.

Detailed descriptions of the methods used are given in the sections below.

**S5. Village Matching Methods**

Village selection was only undertaken within Preah Vihear province, because different provinces are subject to different political and socio-economic factors. In total, 6 villages were located within PVPF and 10 villages were located within KPWS, excluding villages around provincial and district towns. The matching dataset compromised the 208 official villages recorded by the Commune Database (NCDD, 2011), plus 3 unofficial villages of >50 households, a total of 211 villages. Matching procedures are generally more accurate if there are a large number of candidate controls to select from relative to the number of treatment units. The matching variables chosen were: (*1*) Number of families in the village in 2005 from the Commune Database and updated by field surveys; (*2*) Distance to nearest all-weather road in 2005, from the Cambodia Reconnaissance Survey Digital Data (MPWT/JICA, 2003) and updated by field surveys; (*3*) Distance to nearest full-day market in 2005 based on field surveys; and (*4*) Percentage of forest cover within 5 km of the village (the area used regularly by local people) based on the national forest cover assessments from 2005/2006 (Forestry Administration, 2007). The 2005/2006 assessment has an overall classification accuracy of 74%, mainly referring to classification errors between the main forest types (Evergreen Forest, Semi-Evergreen Forest, Deciduous Forest and Other Forest). For the purposes of the matching analysis, all these forest types were combined into a single variable, and expressed as the percentage of land forest cover within 5km of the village within Cambodia. 5km was chosen as the buffer area, because detailed mapping of agriculture around villages within the protected areas indicated that almost all fields are located within this distance of the village (unpublished data). The variables used therefore correspond to the main factors thought to have influenced protected area placement (see Study Sites description), and the main determinants of poverty status at the village level (World Bank, 2009). All the variables selected are exogenous to the interventions being evaluated, i.e. they are not likely to have been affected by the PA interventions prior to 2005. Other similar analyses (e.g. Andam et al. 2008) have included variables relating to soil type and geography (e.g. slope). These variables were not included in this analysis because Preah Vihear is basically flat, with the exception of 3-4 uninhabited hills, and has relatively uniform soils in the study area. It is reasonable to assume that there were no major differences in agricultural and forestry productivity between the PAs and non-PA areas, because no field assessments of productivity were done before the PAs were declared.

Due to the small number of villages inside the protected areas two matches were found for each, to ensure that a sufficiently large number of potential matches were found across the landscape. Analyses were carried out in R 2.14.2 using the package ‘matching’ (Sekhon, 2007; R Development Core Team, 2012). Nearest-neighbor covariate matching was used (Abadie & Imbens, 2006), allowing matching against multiple variables with equal weighting. We preferred covariate matching over propensity score matching because it achieved balance in all four variables and we felt it was easier to understand. The Mahalanobis distance (Abadie & Imbens, 2006) was used to measure distance in the multivariate space, as in previous studies (Andam et al 2008, Andam et al 2010, Joppa & Pfaff, 2010a). All matching was with replacement and ties were handled deterministically by weighting the tied matches (Abadie & Imbens, 2006).

Matching selected 15 possible control villages, and balancing statistics and tests indicated that balance had been achieved in the matched sample (Table S1). Two of the villages were within 20km of the PAs and were excluded from the sample to prevent spillover effects. Random stratified sampling, by district, was then used to select up to 2 matches per district with areas of forest >20 km from the protected area boundaries. This buffer was set to ensure that there were no spillover effects from the protected areas onto the control villages. The random stratified sampling by district ensured that the final control villages selected were distributed across the landscape, rather than being clustered in one area, making the survey design robust in the future against the potential loss of control villages due to large-scale development or other events.

**S6.** Balancing statistics and tests for covariate matching for the unmatched and matched samples of villages inside and outside the protected areas. The matching process ensured that the differences between PA and control villages for the matched sample were not significant. Statistics calculated included the means for each group; the mean, median and maximum difference in the empirical quantile-quantile (Q-Q) plot of treatment and control groups on the scale in which the variable was measured; the variance ratio of treatment over control (which should equal 1 if there is perfect balance); t-tests comparing the samples before and after matching (the two sample t-test was used pre-matching and the paired t-test was used post-matching); and the bootstrap Kolmogorov-Smirnov test, which tests for a significant difference across the entire distribution (as indicated by the empirical Q-Q plots).

| Variable | Village Size (families) | | Distance to all-weather Road (km) | | Distance to full-day Market (km) | | Forest Cover in 2005/6 (%) | |
| --- | --- | --- | --- | --- | --- | --- | --- | --- |
| Statistic | Unmatched | Matched | Unmatched | Matched | Unmatched | Matched | Unmatched | Matched |
| Mean PA villages | 131.2 | 131.2 | 27.6 | 27.6 | 24.3 | 24.3 | 94.7 | 94.7 |
| Mean Control villages | 167.2 | 129.5 | 13.9 | 22.6 | 9.6 | 23.0 | 74.4 | 92.2 |
| Std Deviation Mean diff | -65.9 | 3.0 | 61.0 | 22.1 | 271.9 | 23.6 | 657.0 | 81.4 |
| Mean raw eQQ diff | 90.0 | 17.8 | 15.5 | 8.0 | 14.3 | 1.3 | 20.8 | 2.5 |
| Median raw eQQ diff | 22.0 | 18.0 | 11.6 | 5.1 | 15.3 | 1.0 | 16.5 | 2.6 |
| Max raw eQQ diff | 963.0 | 38.0 | 31.6 | 25.0 | 18.7 | 5.3 | 50.5 | 5.9 |
| Mean eCDF diff | 0.078 | 0.095 | 0.259 | 0.109 | 0.431 | 0.068 | 0.433 | 0.160 |
| Median eCDF diff | 0.065 | 0.100 | 0.235 | 0.100 | 0.464 | 0.067 | 0.474 | 0.133 |
| Max eCDF diff | 0.222 | 0.233 | 0.540 | 0.267 | 0.764 | 0.200 | 0.801 | 0.333 |
| Var ratio (Treatment/Control) | 0.182 | 2.13 | 1.179 | 2.189 | 0.502 | 1.045 | 0.0344 | 0.619 |
| T-test p-value | 0.041 | 0.857 | 0.036 | 0.114 | < 0.001 | 0.055 | < 0.001 | 0.102 |
| KS Bootstrap p-value | 0.408 | 0.310 | <0.001 | 0.194 | < 0.001 | 0.463 | < 0.001 | 0.058 |

**S7.** Balancing statistics and tests for covariate matching for the unmatched and matched samples of squares for (a) Protected Areas and Controls, and (b) areas affected by PES interventions and areas that were not. The matching process ensured that the differences between the PA and Control squares for the matched sample were minimized. Statistics calculated included the means for each group; the mean, median and maximum difference in the empirical quantile-quantile (Q-Q) plot of treatment and control groups on the scale in which the variable was measured; the variance ratio of treatment over control (which should equal 1 if there is perfect balance); t-tests comparing the samples before and after matching (the two sample t-test was used pre-matching and the paired t-test was used post-matching); and the bootstrap Kolmogorov-Smirnov test, which tests for a significant difference across the entire distribution (as indicated by the empirical Q-Q plots).

(a) Protected Areas and Controls

| Variable | Slope (degrees) | | Distance to nearest village (km) | | Forest Cover in 2005/6 (hectares) | |
| --- | --- | --- | --- | --- | --- | --- |
| Statistic | Unmatched | Matched | Unmatched | Matched | Unmatched | Matched |
| Mean PA squares | 1.478 | 1.396 | 4.508 | 4.496 | 95.451 | 95.424 |
| Mean Control squares | 1.914 | 1.400 | 4.232 | 4.493 | 94.978 | 95.491 |
| Std Deviation Mean diff | -32.093 | -0.548 | 15.227 | 0.170 | 4.060 | -0.581 |
| Mean raw eQQ diff | 0.517 | 0.020 | 0.274 | 0.032 | 0.771 | 0.203 |
| Median raw eQQ diff | 0.290 | 0.010 | 0.284 | 0.028 | 0.075 | 0.000 |
| Max raw eQQ diff | 8.783 | 2.394 | 0.432 | 0.302 | 9.429 | 17.757 |
| Mean eCDF diff | 0.140 | 0.007 | 0.042 | 0.005 | 0.058 | 0.007 |
| Median eCDF diff | 0.154 | 0.007 | 0.048 | 0.004 | 0.032 | 0.004 |
| Max eCDF diff | 0.218 | 0.029 | 0.064 | 0.018 | 0.159 | 0.035 |
| Var ratio (Treatment/Control) | 1.099 | 1.108 | 0.943 | 0.995 | 0.981 | 1.036 |
| T-test p-value | <0.001 | 0.243 | <0.001 | 0.539 | 0.346 | 0.039 |
| KS Bootstrap p-value | <0.001 | 0.609 | 0.016 | 0.978 | <0.001 | 0.218 |

(b) PES areas and non-PES areas, all within Protected Areas

| Variable | Slope (degrees) | | Distance to nearest village (km) | | Forest Cover in 2005/6 (hectares) | |
| --- | --- | --- | --- | --- | --- | --- |
| Statistic | Unmatched | Matched | Unmatched | Matched | Unmatched | Matched |
| Mean PES squares | 1.338 | 1.255 | 2.809 | 3.094 | 87.243 | 95.334 |
| Mean non-PES squares | 1.335 | 1.258 | 2.708 | 3.076 | 92.730 | 95.268 |
| Std Deviation Mean diff | 0.676 | -0.786 | 9.871 | 1.966 | -31.870 | 0.539 |
| Mean raw eQQ diff | 0.070 | 0.013 | 0.118 | 0.036 | 5.420 | 0.247 |
| Median raw eQQ diff | 0.065 | 0.009 | 0.116 | 0.031 | 3.568 | 0.000 |
| Max raw eQQ diff | 0.850 | 0.067 | 0.302 | 0.166 | 16.259 | 3.076 |
| Mean eCDF diff | 0.048 | 0.010 | 0.030 | 0.013 | 0.139 | 0.015 |
| Median eCDF diff | 0.042 | 0.008 | 0.031 | 0.008 | 0.151 | 0.008 |
| Max eCDF diff | 0.131 | 0.041 | 0.068 | 0.049 | 0.228 | 0.049 |
| Var ratio (Treatment/Control) | 1.298 | 0.995 | 0.890 | 0.974 | 1.420 | 1.008 |
| T-test p-value | 0.933 | 0.392 | 0.245 | 0.027 | <0.001 | 0.508 |
| KS Bootstrap p-value | 0.011 | 1.000 | 0.486 | 0.998 | <0.001 | 0.973 |

**S8. Deforestation Analysis**

The analysis focused on all 16 villages inside the PAs, 7 controls and 11 villages that bordered the PAs. Landsat and Aster satellite images were used to estimate forest cover at three nominal time points: 2001/2, 2005/6, and 2009/10 (Rainey et al. 2010). Imagery from the Cambodian dry season (December-March) was used as this is likely to show greater contrast between forest and non-forest areas and lower cloud cover. Imagery covering the whole of the landscape in the 2002 dry season was not available, so for some sections imagery was used from a different sensor and from the 2001 and 2003 dry seasons (Table S2). For 2010, the best data were available from Landsat 7 (ETM+), which suffers from scratch lines due to a persistent sensor error. Gaps were filled with temporally adjacent images using standard techniques. The mosaic method was used to combine the various image fragments into one single image for the study area for each year. Two other types of satellite image were often available for each dry season, and these have been used as ancillary data the visual assessments.

Interpretation delineated two vegetation classes, forest and non-forest. The official national definition^^[[2]](#footnote-2)^^ of forest is an area of land covering at least 0.5 ha with at least 10% cover of trees taller than 5 m. This is a slightly lower percentage of crown cover than is used by the Forestry Administration in their assessments (20%; Brun 2009). Non-forest is all land with canopy forest cover less than this, and so includes natural grassland, bare land, water, swidden agriculture, rice paddy, other agricultural land, settlements and deforested areas. Mature tree plantations were not found in the landscape. The minimum mapping unit for forest was 1 ha, due to the limitations of medium-resolution imagery, since an area of 3x3 pixels (90 m x 90 m, 0.81 ha) was the smallest unit that could realistically be identified. This differs slightly from the 0.5 ha criterion used in national definitions but at a landscape level did not result in any bias in deforestation statistics. The image pre-processing and interpretation processing were done using the software package ERDAS IMAGINE. Subsequent data analysis was performed using ArcView 3.3 and ArcGIS 9.3. Images were geometrically corrected to an image from a reference year, which was in turn corrected to the rivers in the national hydrology dataset. A general accuracy of +/-1 pixel was achieved. The same methods gave overall accuracy of 93% when applied in Seima Protection Forest landscape in Mondulkiri which has a similar range of habitats (Evans et al. 2009).

Non-forest patches were identified visually and then delineated using a semi-automatic approach with the “Seed Tool” extension in ArcGIS 9.3. Data from the three sensors (Landsat TM, Landsat ETM+ and ASTER) were analyzed in comparison to high quality ancillary datasets for the whole study area, such as ground-truthed datasets and aerial photographs. For the baseline year, 2001/2, high resolution aerial photographs were available for the entire study area, which clearly showed areas of forest and non-forest. For years after 2002, the non-forest polygons of the previous time point were taken as the baseline and any observed changes were incorporated by editing and recapturing polygon boundaries.

The forest cover maps were resampled using 1km grid squares, to give estimates of the number of hectares of deforestation in the two time periods in each square. Only squares with complete forest cover maps were used. For the analysis of the impacts of PAs, squares were assigned a treatment type depending upon whether they were in a PA and within 8km of one of the 16 villages inside a PA (*n* = 1356), within 8 km of one of the 7 control villages (*n* = 913), or within 8km of one of the 11 villages bordering a PA but completely outside the PA itself (*n* = 1035). The 8km radius was chosen because this was the maximum distance local people travelled to agricultural plots. For the analysis of the impacts of PES within the PAs, squares were assigned a treatment type depending upon whether they were in the PA and within 5km of one of the 4 villages receiving payments (*n* = 217), or were in the PA and within 5km of another village in the PA that was not receiving payments (*n* = 433). The 5km radius was chosen in order to separate out effects due to the different villages within the PAs, where the minimum distance between villages was 10km.

**S9.** Remote sensing data sources used to map forest cover extent in each year.

| **Dry seasons** | **Master image dates** | **Sensor (resolution)** |
| --- | --- | --- |
| 2001/02 | 10 Jan 2002 and 02 Feb 2002  with 17 Feb 2001,19 Jan 2002,  20 Feb 2002, 06 Jan 2003  Aerial Photographs: 2001. Sourced from Department of Geography, Ministry of Land Management, Urban Planning and Construction | Landsat 7 (30 m)  ASTER (30 m)  Photographs (<1m) |
| 2005/06 | 21 Jan 2006  07 Feb 2006  23 Feb 2006 | ASTER (30 m)  Landsat 5 (30 m) |
| 2009/10 | 24 Dec 2009 with 09 Jan 2010*  05 Mar 2010 with 17 Feb 2010*  18 Feb 2010 | Landsat 7 (30 m)  Landsat 7 (30 m)  Landsat 5 (30 m) |

*The second image was used to fill sensor defects in the first image.

**S10. Matching Estimators for deforestation rates**

Matching estimators are widely accepted to be a more robust method for estimating the differences in deforestation rates between two samples, because they adjust for observed differences in the characteristics of the sample areas (Joppa & Pfaff 2010b). The first-level of matching (at the village scale) identified appropriate control village areas to the within-PA group, that were also in remote forested areas. However, other factors might be expected to influence deforestation at the local scale. Deforestation rates are, for example, affected by the amount of forest present and are significantly greater near to population centers or in flat areas. Consequently, a second level of matching was used to select appropriate comparison squares to estimate effects.

The matching variables selected were: (*1*) the base area of forest in hectares in 2001/02, for the first four year period, or 2005/06, for the second four year period; (*2*) the distance in kilometers to the nearest village; and (*3*) the slope in degrees. Elevation, which has been used in similar analyses (Joppa & Pfaff 2010a), was considered but discarded because it was highly correlated with slope, and the area is relatively flat. Similarly soil was not included because soils in the area are relatively uniform and good soil maps are not available.

Matching was conducted six times, to compare the PA squares to the Controls and Border areas in each of the two time periods, and to compare areas within PAs that were affected by PES interventions and areas within PAs that were not affected by PES interventions. Analyses were carried out in R 2.14.2 using the package ‘matching’ (Sekhon 2007; R Development Core Team, 2012). Nearest-neighbor covariate matching was used (Abadie & Imbens, 2006), allowing matching against multiple variables with equal weighting. The Mahalanobis distance (Abadie & Imbens, 2006) was used to measure distance in the multivariate space, as in previous studies (Andam et al 2008, Andam et al 2010, Joppa & Pfaff, 2010a). One match was selected for each grid square inside the PAs or affected by the PES interventions. All matching was with replacement and ties were handled deterministically by weighting the tied matches (Abadie & Imbens, 2006). Calipers were used where necessary to define an acceptable distance for any match, rejecting squares for which matches could not be found, in order that differences in the final sample were minimized. Balancing statistics and tests were used to check that balance had been achieved in the matched samples, and the matching was re-run if the samples were significantly different in two or more characteristics. The balancing statistics and tests for the two most important comparisons are given in Table S3: (a) comparing PAs and the Controls during the second period (2005/6 to 2009/10) and (b) comparing PES areas with non-PES areas within PAs for the second period (2005/6 to 2009/10).

Three methods were used to estimate the difference in deforestation rates: (*1*) the average difference between the deforestation rates in the squares inside the PAs and the other two treatments; (*2*) matching of squares inside the PAs with similar squares in the other two treatments; and (*3*) matching but accounting for spatial autocorrelation by clustering the data according to the closest village using the equations developed by Hanson and Sunderam (2011). Table S4 provides the three sets of estimators.

**S11.** Estimates of the difference in the absolute deforestation rate (in hectares) between Protected Areas and Controls, Protected Areas and Border areas, and around villages inside PAs receiving payments and villages not receiving payments. Positive values indicate that Protected Areas or PES areas had a greater deforestation rate, negative values indicate that Protected Areas or PES areas had a lower deforestation rate. Data is based upon the deforestation rate of 1km grid squares (in hectares). Three estimators are given: the average deforestation rate in each treatment type, the matching estimator calculated without using clustering, and the matching estimator calculated adjusting for the clustering effect due to village. The clustered matching estimator is the most robust because this accounts for the pseudo-replication due to having multiple 1k grid squares around each study village.

| Differences in Deforestation Rates: | 2001/2 to 2005/6 | | 2005/6 to 2009/10 | |
| --- | --- | --- | --- | --- |
|  | Difference | Significance ^a^ | Difference | Significance ^a^ |
| 1. Controls v. Protected Areas |  |  |  |  |
| - average | 0.526 | 2.60 *** | -1.366 | -6.17 *** |
| - matching (unclustered) | -0.139 | -0.93 (ns) | -1.043 | -4.57 *** |
| - matching (clustered) | -0.185 | -0.56 (ns) | -1.152 | -3.95 *** |
| 2. Border Areas v. Protected Areas |  |  |  |  |
| - average | -1.322 | -6.70 *** | -2.959 | -14.60 *** |
| - matching (unclustered) | -0.551 | -2.46 * | -1.964 | -8.12 *** |
| - matching (clustered) | -0.592 | -0.96 (ns) | -2.352 | -3.80 *** |
| 3. No PES v. PES (inside Protected Areas only) |  |  |  |  |
| - average | 1.996 | 4.12 *** | -0.564 | -3.15 *** |
| - matching (unclustered) | 1.503 | 4.88 *** | -0.712 | -4.30 *** |
| - matching (clustered) | 1.446 | 1.94 (ns) | -0.712 | -2.14 * |

^a^ Tests of difference are t-tests for the average difference and unclustered matching and z-tests for clustered matching; t-values, z-values and the significance are given. Significance values: ns = not-significant; * *P* < 0.05; ** *P* < 0.01; *** *P* < 0.001.

**S12. Using the Basic Necessities Survey as a measure of Household Poverty**

Poverty is a multi-faceted concept incorporating multiple dimensions (Scoones, 1998; Sen, 1999; McGregor, 2007), which can be measured in several aspects (Agrawal & Redford, 2006). An important methodological problem in social impact assessment therefore concerns how to define and measure poverty in order to assess trends (Ravallion, 2003). We used the Basic Necessities Survey because it incorporates multiple aspects of poverty into a single score for each household in the sample, relative to a locally-derived definition (Davies & Smith, 1998; Pro-Poor Centre & Davies, 2006). The Basic Necessities Survey is based on previous methods that have been used in both developed and developing countries (Mack & Lansley, 1985; Hallerod, 1994; Noble et al., 2008). Previous research had indicated that the Basic Necessities Survey score was highly correlated with other poverty measures (Clements et al. 2012b).

Basic necessities are defined as assets or services that 50% or more of respondents agree “*are basic necessities that everyone in the community should be able to have and nobody should have to go without*“. During the survey, respondents are asked to choose which items from a list meet the basic necessities definition, and they are then asked if they have the item currently. Items are weighted for importance according to the percentage of respondents who say an item is a basic necessity, discarding items that <50% of people thought met the basic necessity definition. Household poverty scores are based on the sum of the weightings of the basic necessities they have, as a percentage of the sum of the weightings for all basic necessities. The Basic Necessities Survey has the advantage that the population sampled defines the poverty score weightings, i.e. poverty is locally defined. Including a large variety of assets and services in the Basic Necessities Survey list means that the final score captures a wide range of the dimensions of poverty. The Basic Necessities Survey list of assets and services was defined during initial focus group discussions in villages not selected for the full surveys and district or provincial towns.

The list was constructed to contain a mixture of 35 items (assets or services) that included: (*i*) Items everyone in the study area agreed were basic necessities, and the majority of households had (e.g. a knife, having three meals a day); (*ii*) Items everyone in the study area agreed were basic necessities, but only some people had (e.g. draft animals, access to secondary schools, a toilet); (*iii*) Items <50% of people in the study area thought were basic necessities, but which were basic necessities to people in towns (e.g. mobile phones, electricity, a television); (*iv*) Items no one in the study area thought were basic necessities, and which people in towns did not rate as basic necessities, but which might become basic necessities in the future (e.g. health insurance, having a holiday). The list therefore deliberately included items no one would consider necessities, in order to encourage respondents to consider their answers rather than just marking all items as basic necessities. Including items that might become basic necessities as the aspirations change also allowed the same list to be used to measure future trends. Thirty-five items was thought to be an appropriate length for the list in order to keep the questionnaire short.

It was decided that a minimum of 40 households per village should be assessed in order to accurately capture the within-village variation (particularly where there are potentially important differences in the impact of the intervention on households following different livelihood strategies). There was therefore a trade-off in the survey design between interviewing a small number of people in many villages versus a larger number of people in fewer villages. The within-village variation was interesting in this study for two reasons: (*1*) in order to capture the PES impacts, since only a subset of households in each village received payments; and (*2*) in order to capture the within-village variation, such as the differential impacts on resin-tappers.

The first assessment was conducted in September-November 2008, three years after the PA management activities were initiated, and coinciding before the market-linked PES programs were scaled up in four villages. Households were selected using random stratified sampling, based on a participatory wealth ranking exercise in each village. In total, 871 households were interviewed in 2008 (504 within PAs, 205 controls, and 162 in border villages). The second assessment took place in July-September 2011 with the aim of interviewing the original households and an expanded sample of households in the payment villages. Approximately 12% of the original sample could not be located, either because people had moved away or were absent; these were replaced by interviewing a household of a similar wealth status. The total sample in 2011 had 1053 households, including the replacement households and the expanded sample households from payment villages (686 within PAs, 203 controls, and 164 in border villages). Of the expanded within-PA sample, 180 households had received payments during the 2008-2011 period. The final panel had 769 households that were interviewed in both time periods (443 within PAs, 185 controls, and 141 in border villages).

**S13**. Change in household wellbeing and livelihood strategies for a panel of 769 households in the northern Cambodia during 2008-2011, showing changes for the poorest and richest quintiles in 2008. Poverty was measured using the Basic Necessities Survey score.

|  | All | | Bottom Quintile | | Top Quintile | |
| --- | --- | --- | --- | --- | --- | --- |
|  | 2008 | 2011 | 2008 | 2011 | 2008 | 2011 |
| Households | 769 | 769 | 153 | 153 | 151 | 151 |
| Household Size | 5.8 | 5.9 | 5.5 | 5.7 | 6.1 | 6.0 |
| ♯ Working Adults | 3.1 | 3.2 | 2.8 | 2.9 | 3.5 | 3.6 |
| Dependency Ratio | 1.0 | 1.0 | 1.2 | 1.2 | 0.9 | 0.8 |
| Female-headed households (%) | 8 | 7 | 12 | 13 | 3 | 7 |
| Household head Education (years) | 3.0 | 3.1 | 1.8 | 1.7 | 4.7 | 4.8 |
| Household Head Age (years) | 41.1 | 43.4 | 38.2 | 40.9 | 42.5 | 45.0 |
| *Wellbeing Variables* |  |  |  |  |  |  |
| Poverty | 9.4 | 11.8 | 5.0 | 9.0 | 14.0 | 14.4 |
| Rice total harvest (kg) | 1777 | 2557 | 816 | 1355 | 2893 | 3820 |
| Food security (kg) | -244 | 1393 | -972 | 264 | 646 | 2625 |
| Resin yield (liters) |  | 619 |  | 417 |  | 605 |
| *Livelihood Strategies* |  |  |  |  |  |  |
| Resin-tapper (%) | 44 | 49 | 31 | 41 | 50 | 42 |
| Rice Farmer (%) | 92 | 96 | 83 | 94 | 97 | 97 |
| Have >1 hectare of paddyfields (%) | 74 | 85 | 37 | 66 | 97 | 97 |
| Rice Shifting Cultivation | 39 | 29 | 52 | 48 | 20 | 17 |
| Cash Crops (%) |  | 5 |  | 2 |  | 7 |
| Employed (%) | 6 | 8 | 1 | 3 | 14 | 16 |
| - Public Sector (%) | 5 | 5 | 1 | 1 | 11 | 13 |
| Service or Shop (%) | 16 | 26 | 5 | 9 | 38 | 49 |
| *Assets* |  |  |  |  |  |  |
| ♯ Cattle (heads) | 4.1 | 3.5 | 1.7 | 1.8 | 7.3 | 6.2 |
| Mini-tractor (%) | 30 | 54 | 3 | 17 | 64 | 84 |
| Cattle Draft (%) | 37 | 25 | 12 | 22 | 62 | 30 |

**S14.** Change in household wellbeing, livelihood strategies and assets, for the panel of households that decided to participate in the three PES programs between 2008 and 2011, from the same villages within the Protected Areas. Data for the Ecotourism and Ibis Rice program are based upon 4 villages (174 households) where the programs were implemented; data for the Bird Nests program are based upon 6 villages (247 households). Households could have more than one livelihood strategy. Poverty was measured using the Basic Necessities Survey score.

|  | Bird Nests participants | | Ecotourism participants | | Ibis Rice participants | |
| --- | --- | --- | --- | --- | --- | --- |
|  | 2008 | 2011 | 2008 | 2011 | 2008 | 2011 |
| Households | 28 | 28 | 27 | 27 | 50 | 50 |
| Household Size | 5.9 | 6.3 | 6.4 | 6.9 | 6.0 | 6.3 |
| ♯ Working Adults | 3.2 | 3.6 | 4.1 | 4.6 | 3.5 | 3.9 |
| Dependency Ratio | 1.0 | 0.9 | 0.6 | 0.5 | 0.8 | 0.8 |
| Female-headed households (%) | 7 | 11 | 11 | 7 | 0 | 2 |
| Household head education (school years) | 2.7 | 2.9 | 3.8 | 3.9 | 4.0 | 3.8 |
| Household head age (years) | 42.4 | 44.2 | 44.7 | 48.1 | 43.6 | 45.6 |
| *Wellbeing Variables* |  |  |  |  |  |  |
| Poverty | 9.4 | 11.6 | 10.8 | 14.3 | 11.1 | 13.8 |
| Rice Harvest (kg) | 2154 | 2154 | 2811 | 3262 | 2707 | 3697 |
| Food Security (kg) | -194 | 1376 | 304 | 1773 | 486 | 2440 |
| *Household Livelihood strategies* |  |  |  |  |  |  |
| Resin-tapper (%) | 64 | 54 | 56 | 67 | 54 | 62 |
| Rice Farmer (%) | 89 | 93 | 93 | 100 | 96 | 100 |
| Have >1 hectare of paddyfields (%) | 68 | 89 | 93 | 100 | 94 | 96 |
| Rice Shifting Cultivation (%) | 43 | 32 | 11 | 0 | 10 | 2 |
| Employed (%) | 0 | 14 | 19 | 11 | 14 | 18 |
| - Public Sector (%) | 0 | 7 | 15 | 7 | 14 | 12 |
| Shop or Service (%) | 14 | 18 | 19 | 26 | 14 | 38 |
| *Household Assets* |  |  |  |  |  |  |
| Resin yield (liters) | 836 | 441 | 542 | 873 | 528 | 643 |
| ♯ Cattle (heads) | 4.9 | 3.4 | 8.7 | 6.1 | 9.7 | 7.1 |
| Mini-tractor (%) | 29 | 54 | 33 | 85 | 44 | 82 |
| Cattle Draft (%) | 57 | 11 | 48 | 26 | 60 | 34 |

**S15.** Comparison of the characteristics of panel households with an expanded sample of payment households from the same villages in 2011. The two samples are very similar, implying that there was no particular bias in the selection of the panel sample for the key results. Poverty was measured using the Basic Necessities Survey score.

|  | Bird Nests Participants | | Ecotourism Participants | | Ibis Rice  Participants | |
| --- | --- | --- | --- | --- | --- | --- |
|  | Panel | All | Panel | All | Panel | All |
| Households | 28 | 64 | 27 | 50 | 50 | 120 |
| Household Size | 6.3 | 6.1 | 6.9 | 6.2 | 6.3 | 6 |
| ♯ Working Adults | 3.6 | 3.5 | 4.6 | 4.4 | 3.9 | 3.6 |
| Dependency Ratio | 0.9 | 0.9 | 0.5 | 0.5 | 0.8 | 0.8 |
| Female-headed households (%) | 11 | 8 | 7 | 16 | 2 | 5 |
| Household head education (school years) | 44.2 | 44.2 | 48.1 | 47.9 | 45.6 | 44.1 |
| *Wellbeing variables* |  |  |  |  |  |  |
| Poverty | 11.6 | 11.9 | 14.3 | 13.9 | 13.8 | 13.7 |
| Rice Harvest (kg) | 2154 | 2682 | 3262 | 3222 | 3697 | 3450 |
| Food Security (kg) | 1376 | 1542 | 1773 | 1903 | 2440 | 2231 |
| *Household Livelihood strategies* |  |  |  |  |  |  |
| Resin-tapper (%) | 54 | 63 | 67 | 62 | 62 | 63 |
| Rice Farmer (%) | 93 | 94 | 100 | 100 | 100 | 100 |
| Have >1 hectare of paddyfields (%) | 89 | 91 | 100 | 98 | 96 | 98 |
| Rice Shifting Cultivation (%) | 32 | 19 | 0 | 2 | 2 | 3 |
| Employed (%) | 14 | 13 | 11 | 12 | 18 | 13 |
| Shop or Service (%) | 18 | 19 | 26 | 22 | 38 | 23 |
| *Household Assets* |  |  |  |  |  |  |
| Resin yields (liters) | 441 | 915 | 873 | 786 | 643 | 756 |
| ♯ Cattle (heads) | 3.4 | 4.0 | 6.1 | 5.9 | 7.1 | 6.2 |
| Mini-tractor (%) | 54 | 61 | 85 | 84 | 82 | 79 |
| Draft Cattle (%) | 11 | 20 | 26 | 30 | 34 | 31 |

**S16. Mixed Effects Models for Households Survey Data**

The PA impact evaluation used the entire panel of 769 households that were interviewed in 2008 and 2011 (443 within PAs, 185 controls, and 141 in border villages). In addition, the complete sample of households in 2008 (871 households total) and 2011 (1053 households total) were analyzed, in order to see if results were robust. The PES impact evaluations used a subset from villages within the PAs: for Ecotourism and Ibis Rice 174 households from 4 villages, of which 27 and 50 households respectively were paid during 2008-11; and for the Bird Nests 247 households from 6 villages, of which 28 were paid during 2008-11.

The dependent variables used for all analyses of household wellbeing were: (*1*) the Basic Necessities Survey score, as a measure of household poverty; (*2*) rice harvest, since rice is the staple crop in Cambodian diets; (*3*) food security, defined as the difference between harvests and household rice needs; and (*4*) the number of years of education completed by each family member. Households were also categorized by which livelihood strategies they practiced: resin-tappers, rice farmers, having >1 hectare of paddyfields, owning a mini-tractor, doing shifting cultivation or cash crops, having a job, and running a shop or a family business. The impacts of the PA and PES interventions were investigated using mixed effects models, in order to account for the random effect due to village. We investigated the correlation between the random effect of village and the error term, but there was no relationship. Models were formulated for each variable in 2008 and 2011, and for the change in each variable between 2008 and 2011. The latter is called the ‘first difference’ model by economists (Wooldridge, 2002), and eliminates the potential bias due to unobserved factors (e.g. unobserved household or village characteristics) so long as the impact of these factors can be assumed to be similar over time for different treatment units. This assumption was likely to be met for the analysis of the impact of the payment programs, because payment households and controls were drawn from the same villages inside Protected Areas, where any unobserved factors households experienced would probably have been the same. For the analysis of the impact of Protected Areas, it is possible that unobserved factors could have varied in different ways over time for the different villages. However this was minimized in the survey design by ensuring that the villages were as similar as possible (based upon the matching analysis), using a short study period (three years) and by controlling for as many factors as possible in the analysis. First difference models also allow the initial conditions of households – such as baseline poverty status – to be controlled for by including this as a covariate in the model (Wooldridge, 2002). For the purposes of the analyses, all covariate variables (e.g. rice harvests, food security) and their differences were square-root transformed.

Covariate dependent variables (poverty [Basic Necessities Survey score], rice harvests, and food security) were analyzed using mixed effects models in R 2.14.2 (R Development Core Team, 2012) with package “nlme” (Pinheiro et al 2011). Explanatory variables included household characteristics (size, age of members, education levels, etc.), livelihood strategies, agricultural variables, and village-level factors. Variables that were highly correlated were excluded from the same models. Graphical plots were used to investigate possible effects of interactions between the explanatory variables on the two dependent variables. In order to reduce the risk of finding spurious correlations given the large number of possible explanatory variables, competing models were developed based on the *a priori* research hypotheses (Burnham et al., 2011). The initial model fitted contained the main effects of all the key explanatory variables and relevant 2-way interactions based on the main research questions. Second-order AICc values were used to compare competing models, as recommended for cases where the ratio of sample size to the number of parameters being estimated is <40 (Burnham & Anderson, 2002). Interaction terms with AICc ∆ values of >4 (“considerably less” empirical support; Burnham & Anderson, 2002) were removed, with the exception of interactions that were relevant to the research hypotheses (e.g. those involving PA). Models were compared using maximum likelihood estimation, with coefficients for the final models estimated using restricted maximum likelihood (Crawley, 2007). Contrasts in R were used to compare differences between the types of village (Crawley, 2007): (*1*) comparing households inside PAs with households in control villages outside PAs, to evaluate the impact of PAs; and (*2*) comparing households on the border of PAs with households inside PAs. Model validation included plotting the residuals against the fitted values to check for the homogeneity of the variance, against the explanatory variables to check for any unexplained patterns, and histograms of the residuals and normal Q-Q plots to check for normality of the errors.

Binomial categorical variables (e.g. if a household practiced a livelihood strategy) were analyzed using mixed effects models in R package “lme4” (Bates et al. 2011) with a binomial error distribution (logistic regression models). Models were used to compare the differences between interventions and years in terms of the livelihood strategies practiced by households, and to determine which variables were characteristics of households that chose to sign up for PES programs. Education was expressed as whether a child was attending high school or not.

Since some of the original 2008 sample were not found in 2011 it is possible that the panel might have been biased, especially for the PES impact evaluation where the sample sizes were small. Consequently, the characteristics of the panel households were compared with the much larger sample of payment households that was collected in 2011 only.

Table S6 shows the final selected mixed effects model for the effect of the PA and PES interventions, household and village-level characteristics, on the household wellbeing variables for the entire sample of households in 2008 (*n* = 871). Table S7 shows the final selected mixed effects models for the entire sample of households in 2011 (*n* = 871) and Table S8 shows the final selected mixed effects models for the panel of households that were interviewed in both time periods (*n* = 769). These three sets of models provide the basis for the significance values for the wellbeing variables reported in Table 2. Tables S9-S11 show the competing models, based upon AICc ∆ values, for the poverty, rice harvest and food security models given in Table S8.

Table S12 shows the change in the characteristics of the panel of payment households between 2008 and 2011, for households that received payments from the Bird Nests, Ecotourism or Ibis Rice programs during that period. Table S13 compares the characteristics of the panel of payment households with the expanded sample of payment households in 2011. The characteristics of the two samples are very similar, suggesting that there was no particular bias in the panel sample.

**References**

Abadie, A., & Imbens, G. (2006). Large Sample Properties of Matching Estimators for Average Treatment Effects. *Econometrica* 74, 235-267.

Agrawal, A. & Redford, K.H. (2006). *Poverty, development and biodiversity conservation: shooting in the dark?* WCS Working Paper No. 26. Wildlife Conservation Society, New York.

Andam, K.S., Ferraro, P.J., Pfaff, A., Sanchez-Azofeifa, G.A. & Robalino, J.A. (2008). Measuring the effectiveness of protected area networks in reducing deforestation. *Proceedings of the National Academy of Sciences USA*, 105, 16089-16094.

Andam, K.S., Ferraro, P.J., Sims, K.R.E., Healy, A. & Holland, M.B. (2010). Protected areas reduced poverty in Costa Rica and Thailand. *Proceedings of the National Academy of Sciences USA*, 107, 9996-10001.

Arriagada, R.A., Ferraro, P.J., Sills, E.O., Pattanayak, S.K. and Cordero-Sancho, S. (2012) Do payments for environmental services affect forest cover? A farm-level evaluation from Costa Rica. *Land Economics*, *88* (2), 382-399.

Bates, D., Maechler, M. & Bolker, B. (2011) *Package ‘lme4’: Linear mixed-effects models using S4 classes*. R pacjage version 0.999375-39.

Brun, S. (2009). *Elaboration of Cartographic tools for reforestation, CDM and REDD project activities in Cambodia*. ONF International, Paris.

Bruner, A.G., Gullison, R.E. & Balmford, A. (2004). Financial costs and shortfalls of managing and expanding Protected-Area systems in developing countries. *Bioscience*, 12, 1119-1126.

Burnham, K.P. & Anderson, D.R. (2002). *Model selection and multimodel inference: a practical information-theoretic approach*. 2nd Edition. Springer, New York.

Burnham, K.P., Anderson, D.R., & Huyvaert, K.P. (2011). AIC model selection and multimodel inference in behavioral ecology: some background, observations, and comparisons. *Behavioral Ecology and Sociobiology*, 65, 23-35.

Cambodia Daily (2011). More concessions granted in Protected Areas. 6 July 2011. Phnom Penh: Cambodia Daily.

Clements, T., John, A., Nielsen, K., An, D., Tan, S. & Milner-Gulland, E.J. (2010). Payments for biodiversity conservation in the context of weak institutions: Comparison of three programs from Cambodia. *Ecological Economics*, 69, 1283-1291.

Clements, T., Rainey, H.J., An, D., Rours, V., Tan, S., Thong, S., Sutherland, W.J. & Milner-Gulland, E.J. (2012a). An evaluation of the effectiveness of a direct payment for biodiversity conservation: the Bird Nest Protection Program in the Northern Plains of Cambodia. *Biological Conservation*, in press.

Clements, T., Suon, S., An, D., Wilkie, D. & Milner-Gulland, E.J. (2012b) Impacts of Forest conservation policies on local poverty and livelihoods in Cambodia. *World Development,* under review.

Crawley, M.J. (2007). *The R Book*. John Wiley & Sons Ltd, Chichester.

Davies, R., & Smith, W. (1998). *The basic necessities survey: the experience of ActionAid Vietnam*. ActionAid, Hanoi, Vietnam.

Evans, T.D., Hout, P., Phet, P. & Hang, M. (2002). *A study of resin-tapping and livelihoods in Southern Mondulkiri, Cambodia, with implications for conservation and forest management*. Wildlife Conservation Society, Phnom Penh.

Evans, T.D., Heng, B. & Delattre, E. (2009). *Deforestation rates in and around the Seima Biodiversity Conservation Area, Cambodia, 2001-2007*. WCS Cambodia Program, Phnom Penh.

Ferraro, P.J. (2009) Counterfactual thinking and impact evaluation in environmental policy. In: M. Birnbaum & P. Mickwitz (eds.). *Environmental program and policy evaluation*. *New Directions for Evaluation*, *122*, 75-84.

Ferraro, P.J. and Pattanayak, S. (2006) Money for Nothing? A call for empirical evaluation of biodiversity conservation investments. *PLOS Biology*, *4*(4), 482-488.

Forestry Administration (2007). *Final Report: Forest Cover Assessment for year 2005/2006*. Forestry Administration, Phnom Penh.

Hallerod, B. (1994). *Poverty in Sweden: A New Approach to the Direct Measurement of Consensual Poverty*. Umea University, Umea.

Hanson, S.G. & Sunderam, A. (2011). The Variance of Non-Parametric Treatment Effect Estimators in the Presence of Clustering. *The Review of Economics and Statistics*, forthcoming.

Imbens, G.W., and Wooldridge, J.M. (2009) Recent developments in the econometrics of program evaluation. *Journal of Economic Literature*, *47* (1), 5-86.

Joppa, L.N. & Pfaff, A. (2010a). Global protected area impacts. *Proceedings of the Royal Academy of Sciences, Series B*, 278, 1633-1638.

Joppa, L. & Pfaff, A. (2010b) Reassessing the forest impacts of protection: the challenge of nonrandom location and a corrective method. *Annals of the New York Academy of Sciences*, 1185, 135-149.

Mack, J. & Lansley, S. (1985). *Poor Britain*. Allen and Unwin, London.

McGregor, J.A. (2007). Researching wellbeing: from concepts to methodology. In: Gough, I., McGregor, J.A. (eds.), *Wellbeing in Developing Countries: From Theory to Research*. Cambridge University Press, Cambridge.

McKenney, B. & Prom, T. (2002). *Natural Resources and Rural Livelihoods in Cambodia: A Baseline Assessment*. Working Paper 23. Phnom Penh: Cambodia Development Resource Institute.

McKenney, B., Yim, C., Prom, T. & Evans, T. (2004). *Focusing on Cambodia’s High Value Forests: Livelihoods and Management*. Phnom Penh: Cambodia Development Resource Institute and Wildlife Conservation Society.

MPWT/JICA (2003). *Cambodia Reconnaissance Survey Digital Data*. Ministry of Public Works and Transportation/Japanese International Cooperation Agency (MPWT/JICA), Phnom Penh.

NCDD (2011). *Cambodia Commune Database*. National Committee for Sub-National Democratic Development, Phnom Penh. www.ncdd.gov.kh/resources/database/cdb

Noble, M.W.J., Wright, G.C., Magasela, W.K. & Ratcliffe, A. (2008) Developing a Democratic Definition of Poverty in South Africa. *Journal of Poverty*, 11, 117-141.

Pattanayak, S. (2009) *Rough Guide to Impact Evaluation of Environmental and Development Programs*. South Asian Network for Development and Environmental Economics, Kathmandu.

Pinheiro, J. & Bates, D. (2009) Mixed-Effects Models in S and S-PLUS. Statistics and Computing Series, Springer-Verlag, New York, NY, 2000.

Pinheiro, J., Bates, D., DebRoy, S., Sarkar, D., & R Development Core Team (2011). *Nlme: Linear and Nonlinear Mixed Effects Models*. R package version 3.1-101.

Pro-Poor Centre & Davies, R. (2006). *The 2006 Basic Necessities Survey (BNS) in Can Loc District, Ha Tinh Province, Vietnam*. Available at www.mande.co.uk/special-issues/the-basic-necessities-survey/. Accessed on 5 May 2012.

R Development Core Team (2012). *R: A language and environment for statistical computing*. R Foundation for Statistical Computing, Vienna, Austria. http://www.R-project.org/.

Rainey, H., Heng, B, & Evans, T. (2010). *Forest cover trends in the Northern Plains of Cambodia 2002-2010*. WCS Cambodia Program, Phnom Penh.

Ravallion, M. (2003). The debate on globalization, poverty and inequality: why measurement matters. *International Affairs* 79: 739-743.

Ravallion, M. (2006) *Evaluating Anti-Poverty Programs*. Policy Research Working Paper 3625 World Bank, Development Economics Research Group, Washington DC.

Rosenbaum, P. and Rubin, D. (1983) The central role of the propensity score in observational studies for causal effects. *Biometrika*, *70*, 41–55.

Scoones, I. (1998). *Sustainable rural livelihoods: a framework for analysis*. IDS working paper, 72. Institute for Development Studies, Brighton.

Sekhon, J.S. (2007). *Multivariate and Propensity Score Matching Software with Automated Balance Optimization: The Matching Package for R*. Available at http://sekhon.berkeley.edu/papers/MatchingJSS.pdf. Accessed August 10, 2008.

Sen, A.K. (1999). *Development as Freedom*. Oxford University Press, Oxford.

Smith, J., and Todd, P. (2005) Does matching overcome LaLonde’s Critique of nonexperimental estimators? *Journal of Econometrics*, *125* (1-2), 305–53.

Sommerville, M., Jones, J.P.G and Milner-Gulland, E.J. (2009) A revised conceptual framework for Payments for Environmental Services. *Ecology and Society*, *14* (2), 34. www.ecologyandsociety.org/vol14/iss2/art34/

Wooldridge, J.M. (2002). *Econometric Analysis of Cross Section and Panel Data*. MIT Press, Cambridge, Massachusetts.

World Bank. (2009). *Poverty profile and trends in Cambodia, 2007*. *Findings from the Cambodia Socio-Economic Survey.* Report No. 48618‐KH. Poverty Reduction and Economic Management Sector Unit, East Asia and Pacific Region. Washington DC: World Bank.

**S17.** Final mixed effects models of household wellbeing in 2008 only for the entire dataset (*n* = 871). The models show the effect of household and village-level variables in 2008 on (a) household poverty (measured using the Basic Necessities Survey score), (b) household rice harvest and (c) household food security. The table shows the coefficient values for the final model, all of which have a high level of empirical support based on the AICc ∆ values. Part (ii) shows the contrasts tests for significant differences between treatment levels. This table is taken from Clements et al. (2012b, *World Development*).

*(i) Mixed Effects models for effect of interventions on household wellbeing variables in 2008*

| *Coefficient* | (a) Poverty | | (b) Rice Harvest | | (c) Food Security | |
| --- | --- | --- | --- | --- | --- | --- |
| *Impacts of PA Intervention* |  |  |  |  |  |  |
| Intervention [Border PA] | 2.156 |  | 35.633 |  | 106.383 |  |
| Intervention [Inside PA] | 2.223 |  | 29.494 |  | 105.832 |  |
| Intervention [Outside PA] | 2.376 |  | 34.385 |  | 105.830 |  |
| Intervention [Inside PA] : Resin-tapper [Yes] | 0.371 | ns | -1.035 | ns | -1.654 | ns |
| Intervention [Outside PA] : Resin-tapper [Yes] | -0.677 | ns | -6.741 | * | -4.363 | * |
| Intervention [Inside PA] : Own >1 hectare [Yes] |  |  | 4.713 | ns |  |  |
| Intervention [Outside PA] : Own >1 hectare [Yes] |  |  | -2.716 | ns |  |  |
| Intervention [Inside PA] : Own Mini-tractor [Yes] | 1.137 | ** |  |  |  |  |
| Intervention [Outside PA] : Own Mini-tractor [Yes] | 1.301 | ** |  |  |  |  |
| Intervention [Inside PA] : Cattle total (heads) | -0.481 | (*) |  |  |  |  |
| Intervention [Outside PA] : Cattle total (heads) | -0.816 | ** |  |  |  |  |
| *Household Characteristics* |  |  |  |  |  |  |
| Female-headed household [Yes] | 0.922 | ns |  |  |  |  |
| Female-headed household [Yes] * Number of livelihood strategies | -0.613 | * |  |  |  |  |
| Working adults |  |  | 9.085 | *** | 2.340 | * |
| Household size |  |  |  |  | -9.295 | *** |
| Education of household head (years, square-root) | 0.441 | *** |  |  |  |  |
| *Household Livelihood Strategies* |  |  |  |  |  |  |
| Own >1 hectare [Yes] | 1.120 | *** | 5.169 | ns | 3.626 | *** |
| Resin-tapper [Yes] | -0.814 | * | -0.006 | ns | 2.331 | ns |
| Employed [Yes] |  |  | -9.025 | *** | -3.223 | * |
| Rent out labor [Yes] | -0.756 | (*) | -11.759 | *** | -4.187 | * |
| Service provider or Shop-keeper [Yes] | -0.194 | ns | -5.320 | ** |  |  |
| Service provider or Shop-keeper [Yes] * Education of household head (years, square-root) | 0.427 | (*) |  |  |  |  |
| Number of livelihood strategies | 0.887 | *** | 5.041 | *** | 1.452 | ** |
| *Household Assets* |  |  |  |  |  |  |
| Rice Harvest in 2007/8 (kg, square-root) | 0.001 | *** | n/a |  | n/a |  |
| Rice harvest in 2007/8 (kg) * Time to Provincial Capital (hours) | -0.000 | ** | n/a |  | n/a |  |
| Cattle total (heads) | 1.150 | *** | 5.436 | *** |  |  |
| Own Mini-tractor/draft animals [Yes] | 0.233 | ns | 6.512 | * | -0.047 | ns |
| Number of Cattle * Own Mini-tractor/draft animals [Yes] |  |  | -4.130 | ** |  |  |
| Own Mini-tractor [Yes] : Own >1 hectare [Yes] |  |  | 8.891 | *** | 4.620 | *** |
| *Village-level variables* |  |  |  |  |  |  |
| Village Population Size (households) | 0.006 | * | 0.490 | ns |  |  |
| Years of schooling in the village | 0.171 | ns |  |  |  |  |
| Time to Provincial Capital (hours) | -0.079 | ns | -17.092 | (*) | -10.660 | (*) |
| Time to Secondary School (hours) |  |  | -33.812 | * | -16.575 | * |
| Time to Provincial Capital (hours) * Time to Secondary School (hours) |  |  | 13.151 | * | 6.734 | (*) |
| % residual variation due to the random effect of Village | 6.2 | % | 4.3 | % | 4.5 | % |

*(ii) Tests of the differences between interventions for household wellbeing variables in 2008*

| *Contrasts* | (a) Poverty | | (b) Rice Harvest | | (c) Food Security | |
| --- | --- | --- | --- | --- | --- | --- |
| Intervention [Outside PA] > Intervention [Inside PA] | -0.819 | * | -2.027 | ns | -0.912 | ns |
| Intervention [Border PA] > Intervention [Inside PA] | 0.062 | ns | 2.261 | ns | 0.913 | ns |
| Intervention [Outside PA] > Intervention [Inside PA] : Resin-tapper [Yes] | -1.080 | ** | -5.944 | * |  |  |
| Intervention [Outside PA] > Intervention [Inside PA] : Own > 1 hectare [Yes] |  |  | -7.424 | ** | -2.710 | (*) |

**S18.** Final mixed effects models of household wellbeing in 2011 only for the entire dataset (*n* = 1053). The models show the effect of household and village-level variables in 2011 on (a) household poverty (measured using the Basic Necessities Survey score), (b) household rice harvest and (c) household food security. The table shows the coefficient values for the final model, all of which have a high level of empirical support based on the AICc ∆ values. Part (ii) shows the contrasts tests for significant differences between treatment levels.

*(i) Mixed Effects models for effect of interventions on household wellbeing variables in 2011*

| *Coefficient* | (a) Poverty | | (b) Rice Harvest | | (c) Food Security | |
| --- | --- | --- | --- | --- | --- | --- |
| *Impacts of PA and PES Interventions* |  |  |  |  |  |  |
| Intervention [Border PA] | 5.915 |  | 21.264 |  | 76.502 |  |
| Intervention [Inside PA] | 5.315 |  | 20.634 |  | 77.348 |  |
| Intervention [Outside PA] | 5.692 |  | 18.373 |  | 75.756 |  |
| Ibis Rice program, member [Yes] | 0.598 | * | 4.333 | ** | 2.804 | ** |
| Ecotourism program, member [Yes] | 0.912 | * |  |  |  |  |
| Intervention [Inside PA] : Resin-tapper [Yes] | -0.419 | ns | 4.896 | * | 5.168 | * |
| Intervention [Outside PA] : Resin-tapper [Yes] | -1.384 | * | -0.479 | ns | -0.696 | ns |
| Intervention [Inside PA] : Own >1 hectare [Yes] |  |  | -8.581 | * |  |  |
| Intervention [Outside PA] : Own >1 hectare [Yes] |  |  | -3.485 | ns |  |  |
| *Household Characteristics* |  |  |  |  |  |  |
| Female-headed household [Yes] | -0.701 | * | -2.760 | (*) |  |  |
| Working adults |  |  | 5.913 | *** |  |  |
| Household size |  |  |  |  | -2.895 | *** |
| Age of household head (years, square-root) | 1.864 | ns |  |  |  |  |
| Age of household head, squared (years, square-root) | -5.815 | * |  |  |  |  |
| Education of household head (years, square-root) | 0.330 | *** | 1.705 | *** | 1.316 | *** |
| *Household Livelihood Strategies* |  |  |  |  |  |  |
| Rice Farmer Type [None] | 1.116 | * | -38.532 | *** | -12.393 | *** |
| Rice Farmer Type [Shifting Cultivation only] | -0.657 | (*) | -9.397 | *** | -3.970 | ** |
| Rice Farmer Type [Paddy only] | 0.035 | ns | -5.676 | *** | -3.581 | *** |
| Own >1 hectare [Yes] | 1.883 | *** | 10.651 | ** | 3.982 | *** |
| Resin-tapper [Yes] | 0.721 | (*) | -2.169 | ns |  |  |
| Employed [Yes] | 0.863 | ** |  |  | 2.047 | * |
| Service provider or Shop-keeper [Yes] | 1.697 | *** | 2.286 | * | 1.296 | * |
| *Household Assets* |  |  |  |  |  |  |
| Rice Harvest (kg, square-root) | 0.046 | *** | n/a |  | n/a |  |
| Cattle total (heads) | 0.767 | *** | 4.668 | *** | 2.958 | *** |
| Own Mini-tractor [Yes] |  |  | 8.496 | *** | 5.665 | *** |
| *Interactions* |  |  |  |  |  |  |
| Rice Farmer Type [None] : Own Mini-tractor [Yes] |  |  | -19.762 | ** | -14.601 | *** |
| Rice Farmer Type [Shifting Cultivation only] : Own Mini-tractor [Yes] |  |  | -10.382 | * | -7.536 | ** |
| Rice Farmer Type [Paddy only] : Own Mini-tractor [Yes] |  |  | -1.040 | ns | -1.331 | ns |
| % residual variation due to the random effect of Village | 8.7 | % | 4.7 | % | 4.5 | % |

*(ii) Tests of the differences between interventions for household wellbeing variables in 2011*

| *Contrasts* | (a) Poverty | | (b) Rice Harvest | | (c) Food Security | |
| --- | --- | --- | --- | --- | --- | --- |
| Intervention [Outside PA] > Intervention [Inside PA] | -0.013 | ns | -0.609 | ns | -0.215 | ns |
| Intervention [Border PA] > Intervention [Inside PA] | 0.692 | ns | 6.207 | ** | 3.748 | ** |
| Intervention [Outside PA] > Intervention [Inside PA] : Resin-tapper [Yes] | -0.966 | * | -5.036 | * |  |  |
| Intervention [Outside PA] > Intervention [Inside PA] : Own > 1 hectare [Yes] |  |  | 4.548 | (*) | 1.731 | ns |

**S19.** Final mixed effects models for change in household wellbeing between 2008 and 2011 for the panel dataset (*n* = 769). The models show the effect of household and village-level variables on (a) change in household poverty (measured using the Basic Necessities Survey score), (b) change in household rice harvest and (c) change in household food security from 2008 to 2011. The table shows the coefficient values for the final model, all of which have a high level of empirical support based on the AICc ∆ values. Part (ii) shows the tests of difference tests for significant differences between treatment levels. Tables S9-S11 give the model selection tables for the three models shown here.

*(i) Mixed Effects models for the change in household wellbeing variables between 2008 and 2011*

| *Coefficient* | (a) Poverty | | (b) Rice Harvest | | (c) Food Security | |
| --- | --- | --- | --- | --- | --- | --- |
| *Impacts of PA and PES Interventions* |  |  |  |  |  |  |
| Intervention [Border PA] | 5.836 |  | 32.323 |  | 66.315 |  |
| Intervention [Inside PA] | 5.303 |  | 34.646 |  | 62.865 |  |
| Intervention [Outside PA] | 6.236 |  | 29.695 |  | 63.000 |  |
| Ibis Rice program, member [Yes] | 0.712 | * | 5.195 | ** | 3.767 | ** |
| Ecotourism program, member [Yes] | 1.094 | * |  |  |  |  |
| Intervention [Inside PA] : Resin-tapper [Yes] | -0.141 | ns |  |  |  |  |
| Intervention [Outside PA] : Resin-tapper [Yes] | -1.397 | * |  |  |  |  |
| Intervention [Inside PA] : Own >1 hectare [Yes] |  |  | -8.264 | * |  |  |
| Intervention [Outside PA] : Own >1 hectare [Yes] |  |  | -1.088 | ns |  |  |
| *Base Variables* |  |  |  |  |  |  |
| Basic Necessities Survey score in 2008 | -0.608 | *** |  |  |  |  |
| Rice Harvest in 2008 |  |  | -0.799 | *** |  |  |
| Rice Surplus in 2008 |  |  |  |  | -0.846 | *** |
| *Household Characteristics* |  |  |  |  |  |  |
| Female-headed household [Yes] | -0.911 | ** |  |  |  |  |
| Working adults, Change |  |  | 0.827 | * |  |  |
| Household size, Change |  |  |  |  | -0.599 | ** |
| Age of household head (years, square-root) | 4.092 | ns |  |  |  |  |
| Age of household head, squared (years, square-root) | -6.967 | ** |  |  |  |  |
| Education of household head (years, square-root) | 0.209 | * | 2.325 | *** | 1.645 | *** |
| *Household Livelihood Strategies* |  |  |  |  |  |  |
| Rice Farmer Type [None] | -1.276 | * | -38.917 | *** | -12.795 | *** |
| Rice Farmer Type [Shifting Cultivation only] | -0.945 | * | -10.728 | *** | -4.491 | ** |
| Rice Farmer Type [Paddy only] | -0.437 | * | -7.123 | *** | -4.425 | *** |
| Own >1 hectare [Yes] | 2.164 | *** | 8.338 | * | 3.257 | *** |
| Resin-tapper [Yes] | 0.728 | (*) |  |  |  |  |
| Employed [Yes] | 0.856 | ** |  |  |  |  |
| Service provider [Yes] | 1.455 | *** |  |  |  |  |
| Shop-keeper [Yes] | 1.547 | *** |  |  |  |  |
| Service provider or Shop-keeper [Yes] |  |  | 2.421 | * | 1.376 | * |
| *Household Assets* |  |  |  |  |  |  |
| Rice Harvest, change (kg, square-root) | 0.017 | *** | n/a |  | n/a |  |
| Cattle total, change (heads) | 0.419 | *** |  |  | 0.948 | * |
| Own Mini-tractor [Yes] |  |  | 4.060 | ns | 6.916 | *** |
| Own Draft Cattle [Yes] |  |  | 3.901 | ** | 2.038 | ** |
| *Interactions* |  |  |  |  |  |  |
| Own Mini-tractor [Yes] : Own >1 hectare [Yes] |  |  | 7.230 | * |  |  |
| Rice Farmer Type [None] : Own Mini-tractor [Yes] |  |  | 21.817 | ** | -17.581 | *** |
| Rice Farmer Type [Shifting Cultivation only] : Own Mini-tractor [Yes] |  |  | -8.748 | (*) | -7.240 | * |
| Rice Farmer Type [Paddy only] : Own Mini-tractor [Yes] |  |  | -0.449 | ns | -1.055 | ns |
| % residual variation due to the random effect of Village | 10.4 | % | 2.3 | % | 4.3 | % |

*(ii) Tests of the differences between interventions for change in household wellbeing variables between 2008 and 2011*

| *Contrasts* | (a) Poverty | | (b) Rice Harvest | | (c) Food Security | |
| --- | --- | --- | --- | --- | --- | --- |
| Intervention [Outside PA] > Intervention [Inside PA] | 0.417 | ns | 0.829 | ns | 0.135 | ns |
| Intervention [Border PA] > Intervention [Inside PA] | 0.496 | ns | 4.945 | * | 3.450 | * |
| Intervention [Outside PA] > Intervention [Inside PA] : Resin-tapper [Yes] | -1.257 | ** |  |  |  |  |
| Intervention [Outside PA] > Intervention [Inside PA] : Own > 1 hectare [Yes] |  |  | 7.176 | * |  |  |

**S20.** The selected model for the effect of household and village-level variables on the change in household poverty (measured using the Basic Necessities Survey score) during 2008-2011. The table shows (a) the change in AICc caused by removing each term from the model; (b) the change in AICc caused by adding other main effects to the model; and (c) change in AICc for adding other interactions with the PA intervention.

Selected model:

*Interventions:*  PA type [Border/Inside/Outside PA], Ibis Rice program member, Ecotourism program member

*Base Variable:* Poverty in 2008, measured using the Basic Necessities Survey score

*Household Characteristics:* Female-headed [Yes/No], Age of Household Head (squared), Education level of Household Head (in years)

*Household Livelihood Strategies:* Rice Farmer Type [Paddy/Shifting Cultivation/Both/Neither], Own >1 hectare [Yes/No], Resin-tapper [Yes/No], Employed [Yes/No], Provide a Service [Yes/No], Operate a Shop [Yes/No]

*Household Assets:* Change in Rice Harvest (kg), Change in Number of Cattle (heads)

*Interactions with PA intervention:* PA Type * Resin-tapper [Yes/No]

*Other Interactions:* none

*Random Effects:* Village

| **Models** | **K^a^** | **AICc** ∆ |
| --- | --- | --- |
| *(a) Change in AICc for removing each term from the selected model* | | |
| - Ibis Rice program, member | 23 | 1.60 |
| - Ecotourism program, member | 23 | 3.99 |
| - PA Type [Inside/Outside/Border PA] * Resin-tapper [Yes/No] | 20 | 2.56 |
| - BNS Score in 2008 | 23 | 268.67 |
| - Female-headed household [Yes/No] | 23 | 5.51 |
| - Education level of household head (in school years completed) | 23 | 3.73 |
| - Age of household head (squared function) | 22 | 7.09 |
| - Resin-tapper [Yes/No] and interactions | 21 | 6.11 |
| - Own >1 hectare [Yes/No] | 23 | 66.43 |
| - Rice Farmer Type [Paddy/Shifting Cultivation/Both/Neither] | 21 | 4.49 |
| - Rice Harvest, change from 2008-2011 (kg) | 23 | 10.72 |
| - Provide a Service [Yes/No] | 23 | 40.61 |
| - Employed [Yes/No] | 23 | 4.95 |
| - Operate a Shop [Yes/No] | 23 | 23.62 |
| - Number of Cattle, change from 2008-2011 (heads) | 23 | 10.31 |
| *(b) Change in AICc for adding other main effects to the most conservative model* | | |
| + Bird Nest program, member | 25 | 2.12 |
| + Change in Household size (individuals) | 25 | -0.85 |
| + Change in number of Working Adults (individuals) | 25 | 0.14 |
| + Travel time to Provincial Capital, change (hours) | 25 | -0.39 |
| + Travel time to full-day Market, change (hours) | 25 | 2.09 |
| + Village Population, change (number of households) | 25 | 2.11 |
| + Education level available in the village, change (number of school years) | 25 | 2.12 |
| *(c) Change in AICc for adding interactions between livelihood strategies with PA Type* |  |  |
| + PA Type * Female-headed household [Yes/No] | 26 | -1.33 |
| + PA Type * Own >1 hectare [Yes/No] | 26 | 3.78 |
| + PA Type * Rice Farmer Type [Paddy/Shifting Cultivation/Both/Neither] | 30 | 6.45 |
| + PA Type * Provide a Service [Yes/No] | 26 | 3.31 |
| + PA Type * Operate a Shop [Yes/No] | 26 | 2.86 |
| + PA Type * Employed [Yes/No] | 26 | 1.29 |
| + PA Type * Rice Harvest, change from 2008-2011 (kg) | 26 | 1.32 |
| + PA Type * Number of Cattle, change from 2008-2011 (heads) | 26 | 1.04 |

**^a^ K** = number of parameters in the model

**S21.** The selected model for the effect of household and village-level variables on the change in household Rice Harvests during 2008-2011. The table shows (a) the change in AICc caused by removing each term from the model; (b) the change in AICc caused by adding other main effects to the model; and (c) change in AICc for adding other interactions with the PA intervention.

Selected model:

*Interventions:*  PA type [Border/Inside/Outside PA], Ibis Rice program member

*Base Variable:* Rice Harvest in 2008 (kg)

*Household Characteristics:* Change in Number of Working Adults, Education level of Household Head (in years)

*Household Livelihood Strategies:* Rice Farmer Type [Paddy/Shifting Cultivation/Both/Neither], Own >1 hectare [Yes/No], Provide a Service or Operate a Shop [Yes/No]

*Household Assets:* Own a Mini-Tractor [Yes/No], Own Draft Livestock [Yes/No]

*Interactions with PA intervention:* PA Type * Own >1 hectare [Yes/No]

*Other Interactions:* Own >1 hectare [Yes/No] * Own a Mini-Tractor [Yes/No], Rice Farmer Type [Paddy/Shifting Cultivation/Both/Neither] * Own a Mini-Tractor [Yes/No]

*Random Effects:* Village

| **Models** | **K^a^** | **AICc** ∆ |
| --- | --- | --- |
| *(a) Change in AICc for removing each term from the selected model* | | |
| - Ibis Rice program, member | 21 | 5.34 |
| - PA Type [Inside/Outside/Border PA] * Own > 1 hectare [Yes/No] | 18 | 8.55 |
| - Rice Harvest in 2008 | 21 | 548.95 |
| - Education level of household head (in school years completed) | 21 | 25.28 |
| - Change in number of Working Adults (individuals) | 21 | 2.79 |
| - Own >1 hectare [Yes/No] and interactions | 18 | 15.66 |
| - Agriculture Type [Paddy/Shifting Cultivation/Both/Neither] and interactions | 16 | 218.62 |
| - Provide a Service or Operate a Shop [Yes/No] | 21 | 2.84 |
| - Own a Mini-tractor [Yes/No] and interactions | 17 | 81.88 |
| - Own Draft Livestock [Yes/No] | 21 | 9.06 |
| - Own >1 hectare [Yes/No] * Own a Mini-Tractor [Yes/No] | 21 | 3.11 |
| - Rice Farmer Type [Paddy/Shifting Cultivation/Both/Neither] * Own a Mini-Tractor [Yes/No] | 19 | 4.60 |
| *(b) Change in AICc for adding other main effects to the most conservative model* | | |
| + Bird Nest program, member | 23 | 2.07 |
| + Ecotourism program, member | 23 | 1.54 |
| + Change in Poverty (Basic Necessities Survey score) | 23 | 2.11 |
| + Change in Household size (individuals) | 23 | -0.27 |
| + Female-headed household [Yes/No] | 23 | 1.91 |
| + Age of Household Head (years, squared function) | 24 | 0.52 |
| + Resin-tapper [Yes/No] | 23 | 1.04 |
| + Employed [Yes/No] | 23 | 1.62 |
| + Number of Cattle, change from 2008-2011 (heads) | 23 | -0.17 |
| + Travel time to Provincial Capital, change (hours) | 23 | 1.90 |
| + Travel time to full-day Market, change (hours) | 23 | 1.53 |
| + Village Population, change (number of households) | 23 | 1.97 |
| + Education level available in the village, change (number of school years) | 23 | 1.58 |
| *(c) Change in AICc for adding interactions between livelihood strategies with PA Type* |  |  |
| + PA Type * Female-headed household [Yes/No] | 25 | 4.29 |
| + PA Type * Change in number of Working Adults | 24 | -1.05 |
| + PA Type * Rice Farmer Type [Paddy/Shifting Cultivation/Both/Neither] | 28 | 4.26 |
| + PA Type * Resin-tapper [Yes/No] | 25 | 1.76 |
| + PA Type * Provide a Service or Operate a Shop [Yes/No] | 24 | -1.09 |
| + PA Type * Employed [Yes/No] | 25 | 5.61 |
| + PA Type * BNS Score, change from 2008-2011 | 25 | 6.12 |
| + PA Type * Number of Cattle, change from 2008-2011 (heads) | 25 | 2.54 |

**^a^ K** = number of parameters in the model

**S22.** The selected model for the effect of household and village-level variables on the change in household Food Security during 2008-2011. The table shows (a) the change in AICc caused by removing each term from the model; (b) the change in AICc caused by adding other main effects to the model; and (c) change in AICc for adding other interactions with the PA intervention. Food security was defined as the difference between a household’s rice harvest (from paddy and shifting cultivation) minus the household’s food needs for a year.

Selected model:

*Interventions:*  PA type [Border/Inside/Outside PA], Ibis Rice program member

*Base Variable:* Food Security in 2008 (kg)

*Household Characteristics:* Change in Household Size (individuals), Education level of Household Head (in years)

*Household Livelihood Strategies:* Rice Farmer Type [Paddy/Shifting Cultivation/Both/Neither], Own >1 hectare [Yes/No], Provide a Service or Operate a Shop [Yes/No]

*Household Assets:* Own a Mini-Tractor [Yes/No], Own Draft Livestock [Yes/No], Change in Number of Cattle (heads)

*Interactions with PA intervention:* none

*Other Interactions:* Rice Farmer Type [Paddy/Shifting Cultivation/Both/Neither] * Own a Mini-Tractor [Yes/No]

*Random Effects:* Village

| **Models** | **K^a^** | **AICc** ∆ |
| --- | --- | --- |
| *(a) Change in AICc for removing each term from the selected model* | | |
| - Ibis Rice program, member | 19 | 7.04 |
| - PA Type [Inside/Outside/Border PA] | 18 | 3.73 |
| - Food Security in 2008 (kg) | 19 | 531.60 |
| - Change in number of Household Size (individuals) | 19 | 7.58 |
| - Education level of household head (in school years completed) | 19 | 30.26 |
| - Own >1 hectare [Yes/No] | 19 | 10.73 |
| - Agriculture Type [Paddy/Shifting Cultivation/Both/Neither] and interactions | 14 | 93.74 |
| - Provide a Service or Operate a Shop [Yes/No] | 19 | 1.63 |
| - Own a Mini-tractor [Yes/No] and interactions | 16 | 72.05 |
| - Own Draft Livestock [Yes/No] | 19 | 5.47 |
| - Rice Farmer Type [Paddy/Shifting Cultivation/Both/Neither] * Own a Mini-Tractor [Yes/No] | 19 | 3.35 |
| *(b) Change in AICc for adding other main effects to the most conservative model* | | |
| + Bird Nest program, member | 21 | 1.70 |
| + Ecotourism program, member | 21 | 1.83 |
| + Change in Poverty (Basic Necessities Survey score) | 21 | 1.57 |
| + Female-headed household [Yes/No] | 21 | 2.00 |
| + Age of Household Head (years, squared function) | 22 | 3.94 |
| + Resin-tapper [Yes/No] | 21 | 1.86 |
| + Employed [Yes/No] | 21 | 0.08 |
| + Travel time to Provincial Capital, change (hours) | 21 | 2.09 |
| + Travel time to full-day Market, change (hours) | 21 | 0.26 |
| + Village Population, change (number of households) | 21 | 2.01 |
| + Education level available in the village, change (number of school years) | 21 | 2.09 |
| + Own >1 hectare [Yes/No] * Own a Mini-tractor [Yes/No] | 21 | 0.08 |
| *(c) Change in AICc for adding interactions between livelihood strategies with PA Type* |  |  |
| + PA Type * Female-headed household [Yes/No] | 23 | 5.04 |
| + PA Type * Rice Farmer Type [Paddy/Shifting Cultivation/Both/Neither] | 26 | 4.90 |
| + PA Type * Resin-tapper [Yes/No] | 23 | 4.85 |
| + PA Type * Provide a Service or Operate a Shop [Yes/No] | 22 | 2.14 |
| + PA Type * Employed [Yes/No] | 23 | 4.14 |
| + PA Type * BNS Score, change from 2008-2011 | 23 | 5.51 |
| + PA Type * Number of Cattle, change from 2008-2011 (heads) | 22 | 1.73 |

**^a^ K** = number of parameters in the model

1. The Critical Ecosystems Partnership Fund is a joint initiative of l’Agence Française de Développement, Conservation International, the Global Environment Facility, the Government of Japan, the MacArthur Foundation and the World Bank. A fundamental goal is to ensure civil society is engaged in biodiversity conservation. [↑](#footnote-ref-1)
2. As submitted to the United Nations Framework Convention on Climate Change under the Kyoto Protocol’s Clean Development Mechanism. [↑](#footnote-ref-2)
